# The local medicinal plant knowledge in Kashmir Western Himalaya: a way to foster ecological transition via community-centred health seeking strategies

**DOI:** 10.1186/s13002-023-00631-2

**Published:** 2023-11-30

**Authors:** Muhammad Manzoor, Mushtaq Ahmad, Muhammad Zafar, Syed Waseem Gillani, Hamayun Shaheen, Andrea Pieroni, Abdullah Ahmed Al-Ghamdi, Mohamed Soliman Elshikh, Saddam Saqib, Trobjon Makhkamov, Khislat Khaydarov

**Affiliations:** 1 Department of Plant Sciences, Quaid-i-Azam University, Islamabad, Pakistan; 2 State Key Laboratory of Plant Systematics and Evolutionary Botany, Institute of Botany Chinese Academy of Sciences, Beijing, China; 3 Department of Botany, University of Azad Jammu & Kashmir, Muzaffarabad, 13100 Pakistan; 4 University of Gastronomic Sciences Pizza V. Emanuele II, 12042 Pollenzo, Bra, Italy; 5 Department of Botany and Microbiology, College of Sciences, King Saud University, Riyadh, Saudi Arabia; 6 University of Chinese Academy of Sciences, Beijing, China; 7 Department of Forestry and Landscape Design, Tashkent State Agrarian University, 2 A., Universitet Str., Kibray District, 100700 Tashkent Region, Uzbekistan; 8 Institute of Biochemistry, Samarkand State University, Samarkand, Uzbekistan; 10 Department of Medical Analysis, Tishk International University, Erbil, 44001 Kurdistan, Iraq

**Keywords:** Biodiversity hotspot, Endemic, Ethnoecological knowledge, Future conservation, Indigenous communities, Kashmir, Sustainable utilisation

## Abstract

**Background:**

The mountainous region of Kashmir is a biodiversity hotspot, with diverse local communities and a rich cultural history linked to nature. Mountain ecosystems are highly vulnerable to climate change. This study emphasises the need to record the indigenous ethnoecological knowledge of wild plants used for the treatment of various ailments at higher elevations in remote areas where globalisation poses a threat to this traditional knowledge.

**Methods:**

The field survey was carried out in 2020–2022, to collect data on wild medicinal plants. Informants were selected randomly to collect indigenous medicinal knowledge using semi-structured interviews and group discussions. Various quantitative indices were employed to evaluate ethnomedicinal data.

**Results:**

A total of 110 medicinal plants belonging to 49 families were recorded in the study area. These medicinal plants are extensively used by local communities for the treatment of 20 major disease categories. Asteraceae was the dominant family contributing (9.09%) to medicinal plants, followed by Polygonaceae (8.18%), Apiaceae (7.27%), Lamiaceae (5.45%), and Ranunculaceae (5.45%). We observed 166 remedies were used for the treatment of various diseases in humans, and 9 remedies were used for animals. The most frequently used medicinal remedy was tea or decoction (30.91%). Among the medicinal plants, herbs (85.5%) were most frequently used by the local populations of Kashmir, whereas leaves (10.26%) were used for the treatment of various ailments. Out of 110 species, 31 were endemic, 15 of which are endemic to the Kashmir region and 16 to the Western Himalaya. The highest RFC value was reported for *Allium humile (0.77), the* highest UV value for *Fritillaria cirrhosa* (1.33), and the highest ICF value for gastro-intestinal/digestive disorders (0.85).

**Conclusions:**

Local communities still rely on wild medicinal plants for primary healthcare. These communities retained valuable indigenous knowledge, which needs to be preserved for the conservation and sustainable utilisation of natural resources. Further field exploration is required to fully explore indigenous knowledge in the mountainous regions of Kashmir, and this knowledge has the potential to support the ongoing ecological transition.

## Background

The Himalayan Mountain region is home to a diverse range of medicinal and food plants and is regarded as a significant hub of biocultural diversity or biocultural refugia [1]. Particularly, the indigenous communities of the geographical region are more dependent on non-timber forest products, as they derive their livelihood from plant-derived components that are immensely important to the management of traditional healthcare systems [2, 3]. Plants play an essential role in the lives of indigenous peoples living in the Himalayan Mountains, as they provide both food and medicine [2, 4]. Traditional knowledge is the subject of discussion by current ethnobiologist as a result of rapid globalisation and modernisation. According to studies, the remarkable shift in culture has prompted traditional ecological knowledge to decline, if not disappear entirely [5].

Ethnobotanical documentation explores how communities interact with their surroundings, including traditions and cultural beliefs concerning how to utilise certain plants [6–8]. These surveys are important for uncovering novel medicines and highlighting the significance of native medicinal plants [9]. Indigenous communities hold important knowledge about how to utilise a plant for multiple purposes and are custodians of traditional knowledge. This traditional knowledge has been passed down through generations, and it is closely linked to their daily routines and the ecological resources in their surroundings [10]. Indigenous communities struggle to preserve their traditional knowledge, which leads to the need for comprehensive evaluation of this knowledge for better healthcare in isolated areas, as recognised by national as well as global organisations [11].

Despite its significance, indigenous medicinal knowledge is diminishing. Further investigation is required to document medicinal plants, analyse their components, perform clinical trials, and develop novel medications [12, 13]. Ethnomedicine emphasises the therapeutic properties of plants for the development of modern medications. This played a vital role in the development of several pharmaceuticals and modern drug discovery [14]. Approximately 70,000 plant species are used globally as traditional medicine [15], with developing nations depending on plant-based therapies since they are more affordable and safer than conventional therapies, which might be inaccessible [16].

Mountain’s ecosystems in the Himalayan region are essential for economic development and human well-being because they provide a wide range of essential necessities, including fresh water, energy, food, and medicinal plants [17]. The medicinal plants collected by the Ayurvedic physician from the high-altitude area constitute 35.7% of all plant species in the Himalayan alpine and subalpine regions [18]. In the Himalayan region, different ethnic groups have unique indigenous healthcare systems. Depending on the topography and ecology of their various regions, these groups have different uses for medicinal plants [19].

Throughout history, numerous cultures all over the world have utilised medicinal plants as their main source of medicine. Pakistan has an abundance of medicinal and aromatic plants due to its unique phytogeography and diverse climatic conditions. Pakistan is estimated to be home to between 400 and 600 of the world's 5,700 medicinal plants, reflecting the country's rich floral diversity [20]. In the early 1950s, nearly eighty per cent of the population relied on conventional medicinal products for their healthcare requirements [21]. However, this method is currently utilised solely in remote regions as a result of urbanisation and industrialisation [22, 23]. In the Himalayan Mountain region, 70% of medicinal plants and animals are wild, and 70–80% of the local population still relies on traditional remedies for their healthcare [24].

Ethnoecology focuses on the documentation of traditional ecological knowledge, which includes culture and generational ideas passed down through cultural transmission [25]. Ethnoecological knowledge can help with long-term management and conservation of biodiversity, including wild medicinal plants [26–29]. Ethnobotany is beneficial to the development of healthcare and conservation programmes worldwide by preserving and promoting future medicinal plant research for the development of novel medications [30]. Traditional remedies are also widely used for primary healthcare management in developing countries [31] and are in demand in developed countries due to the belief that “natural is better” [32].

Documentation of traditional knowledge is very essential to conserve and enable future research on the safety and effectiveness of medicinal plants, which can validate their traditional use [33]. Traditional medicinal knowledge is generally passed down orally by elderly folks and hakims [34]. The risks of knowledge loss are raised by urbanisation, modern healthcare, and generational gaps [35, 36]. Documenting indigenous ethnomedicinal knowledge is essential for cultural preservation, drug development, and natural resource management. This project explored the potential uses of the data within the realm of domestic healthcare among the local communities living in the Kashmir mountainous areas of the Western Himalayas. The main objective of this study: (1) to document the local ecological knowledge and practices (LEK) linked to medicinal plants in the Western Himalayan region of Kashmir, (2) to compare this LEK with that previously recorded in Pakistan in order to explore data novelty, and (3) to reflect upon if and how the main findings could help to foster sustainability in local health seeking strategies.

## Materials and methods

### Study area

**Fig. 1:**
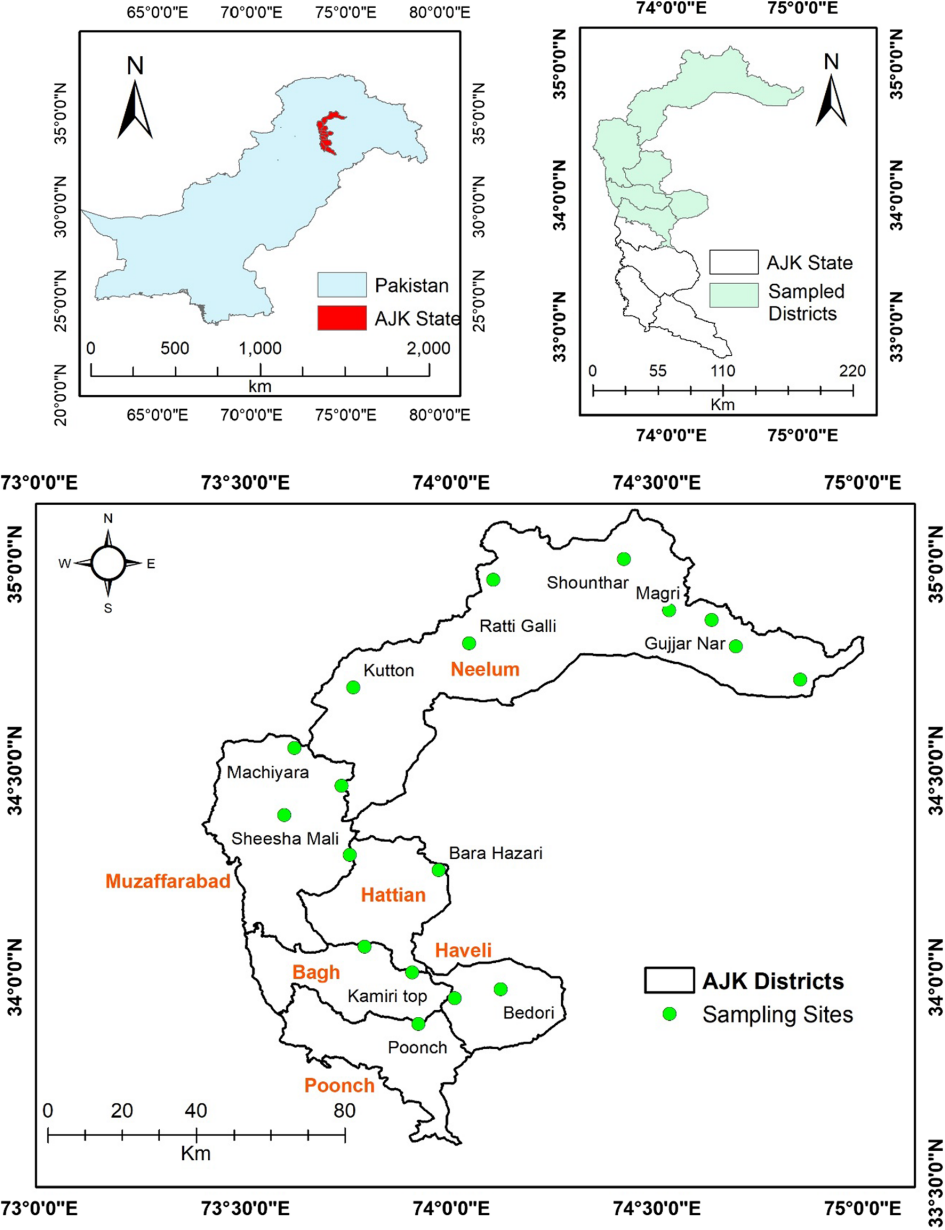
Map of the study area

**Fig. 2:**
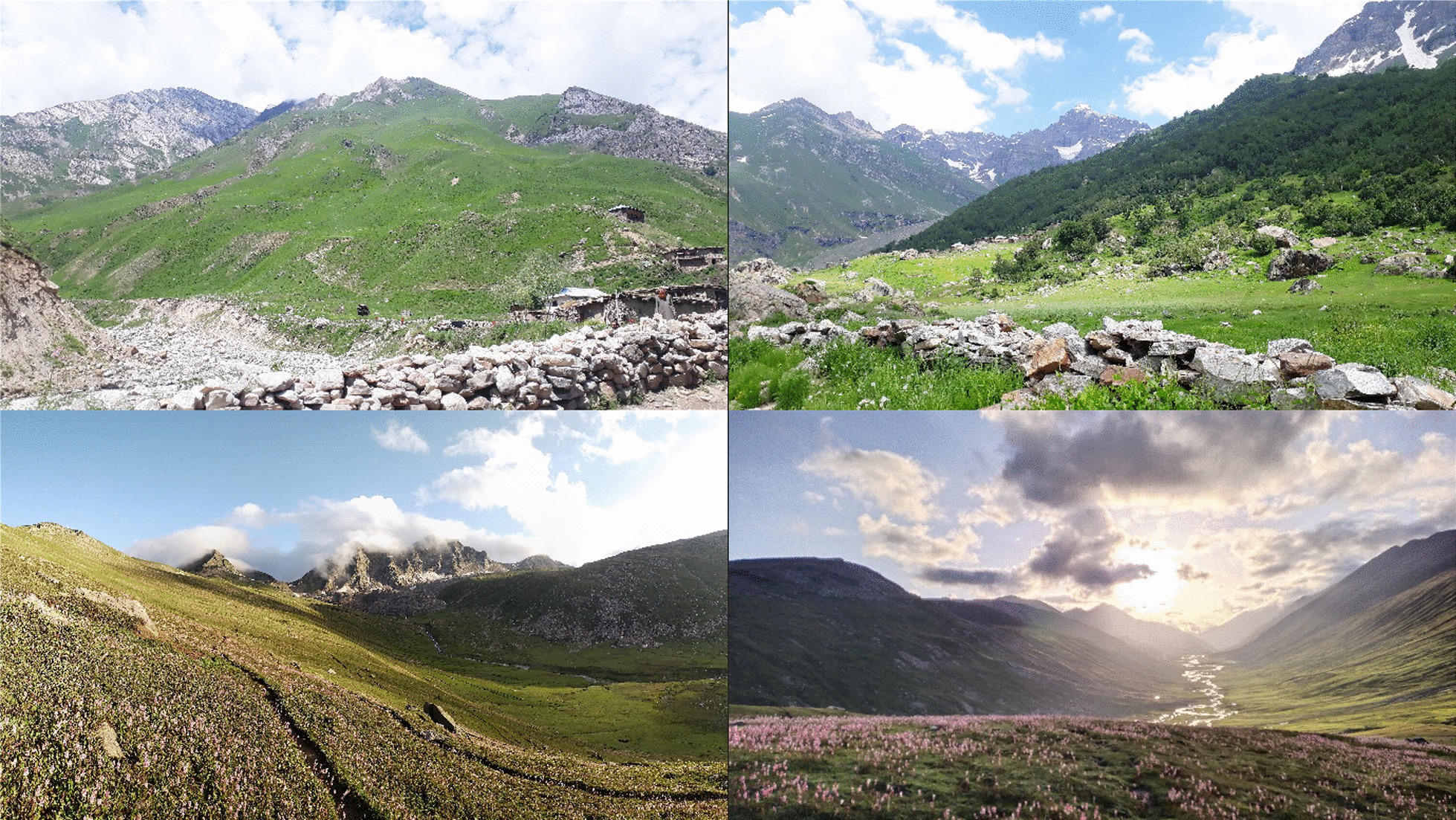
Landscape view of the study area

The State of Azad Jammu and Kashmir (AJK) is located in the Pir-Panjal Subrange of the Western Himalayan Mountains in north-western Pakistan, spread between longitudes 73° and 73° north and latitudes 33° and 36° east, comprising an area of 13,297 km^2^ [37]. The state has a hilly, mountainous topography with forested mountain slopes and deep valleys gorged by several streams and rivers. AJK is a regional biodiversity hotspot harbouring a diverse array of agroclimatic zones and habitats owing to a huge altitudinal gradient ranging from 360 m in the southern parts bounded by the Punjab Plains to an extreme height of 6325 m in the north [38]. An ethnobotanical survey was carried out in the six districts of Kashmir at higher elevations, including Neelum Valley, Jhelum Valley, Muzaffarabad, Haveli, Bagh, and Poonch (Fig. 1). The targeted area consists of subalpine and alpine pastures with an elevational range above 2800 m (Fig. 2). The climate of the study area is characterised by extreme cold during the winter season, with heavy snowfall and freezing temperatures down to − 10 °C from November to April. The average temperature is around 10 °C during the summer from June to August, while the summer season is cold and short. The area receives about 1000 mm of precipitation annually, most of which falls as snow in winter [39].

### Sampling techniques and sampling intensity

In the study area, the multistage random sampling method of [40] was applied for the selection of summer pasture sites. Eighteen summer pasture sites, including Bilor Kasi, Zargary Behak, Gujjar Nar, Shounthar, Magri, Ratti Galli, Dabran top, Peera Hashimar, Sheesha Mali, Bara Hazari, Machiyara, Kamiri top, Sheero Dhara, Bedori, Poonch, Ganga Choti, Saral Behak, and Kutton top with 5% sampling intensity, were selected from the study area. A total of 200 informants were surveyed for documentation of indigenous ethnomedicinal knowledge by the respondents, preferably older people. Furthermore, these respondents had good experience and knowledge regarding the diversity and utilisation of medicinal plants in the study area for primary healthcare management.

### Data collection

About three years (June 2020–September 2022) of field studies were conducted to document the information about ethnomedicinal plants at higher elevations in the study area. Before collecting ethnomedicinal data, each respondent was briefed on the objectives and purposes of the research to gain their consent and cooperation, following the ISE code of ethics (International Society of Ethnobiology, Code of Ethics. 2006) (https://www.ethnobiology.net). The discussions emphasised the importance of each informant's contribution to the record of ethnomedicinal knowledge of medicinal plants in the western Himalayan region of Kashmir. The ethnomedicinal knowledge was documented using open-ended, semi-structured, and pre-tested questionnaire methods. Interviews and group discussions on the indigenous uses of plant species as medicine were used to collect information. Following a preliminary analysis, a group of individuals was chosen, and information was gathered about their interests and abilities in identifying and using plants. The discussions were conducted in the local languages (Pahari, Gojri, or Kashmiri) to facilitate communication with the informants. The information gathered included the local names of medicinal plants, their habits (wild or cultivated), the plant parts used, ethnomedicinal use, the common diseases that can be treated by medicinal plants, the methods of crude drug preparation, and the mode of administration. During field trips, all plants were not in the flowering and fruiting stage. In these instances, data were collected, and the same area was visited during the flowering season.

### Plants identification and preservation

The collected specimens were dried, pressed, and then mounted on the herbarium sheets by following standard protocols [41]. All specimens were identified by Dr Mushtaq Ahmad (Plant Taxonomist) by using available taxonomic literature [42] and online databases of regional flora. The voucher specimens were deposited in the Herbarium of Pakistan (ISL), Quaid-I-Azam University, Islamabad, Pakistan. The endemic status was assessed using online flora (www.efloras.org/flora). Endemic species were classified into two categories: (1) endemic to Kashmir (restricted geographical distribution range) and (2) endemic to the Western Himalayas (broad geographical distribution).

## Data analysis

### Relative frequency of citation

The relative frequency of citation (RFC) was used to analyse ethnobotanical. RCF values measured the local importance of plant species based on the frequency of citation (FC), divided by the total number of informants (N) in the survey. The higher value of the relative frequency of citation (RFC) demonstrates the significance of each species [43].

RFC is calculated using the formula:

$${\text{RFC}} = {\text{FC}}/N$$

### Use value

The use value (UV) measures the relative importance of regionally known plants [44]. The use value is calculated using the formula:

$${\text{UV}} = \Sigma {\text{Ui}}/N$$

where *N* represents the total number of informants and Ui represents the total number of uses claimed by each informant for a particular species.

### Informant consensus factor

The informant consensus factor (ICF) measures if traditional informants consistently used the same plant species [45]. ICF is calculated based on the indigenous information of the informant using a formula:

$${\text{ICF}} = {\text{Nur}} - {\text{Nt}}/{\text{Nur}} - 1$$

where Nur is the total number of reports for all cases of each disease category and Nt is the total number of plant species used in that category. ICF values range 0–1.

### Jaccard index (JI)

To calculate similarities or resemblances of indigenous traditional knowledge data with previous ethnobotanical studies conducted in different areas of the Himalayan region [46], the JI is calculated using the formula:

$${\text{JI}} = \frac{C*100}{{\left( {A + B} \right) - C}}$$

where “*A*” represents the recorded species of the current study and “*B*” is the recorded species of the other area to be compared, while “*C*” is the common number of species in both studies.

## Results and discussion

### Demography of informants

A total of 200 informants, including 137 males (< 45 years = 60.58%, < 30 years = 27.73%, < 18 years = 11.67%) and 63 females (< 40 years = 61.90%, < 18 years = 38.09%), were interviewed in local languages including Phari, Hindko, Gojri, Kashmiri, and Urdu. The majority of female informants were illiterate (Table 1). Female informants’ percentage is lower due to social setup because local people don’t allow women to talk with strangers. The local people of the Kashmir region have a close interaction with nature and the best experience of resource utilisation [47].

**Table 1:** Demographic information of the informants from the investigated area

Summer pasture name	Altitude (m)	Ecology	Stay duration (months)	Ethnicity	Religion	Language	No. of inhabitants	No. of male and female interviewed	Occupation
Bilor Kasi	3517	Alpine pasture	3.5	Kashmiri	Islam	Kashmiri & Hindko	57	8M/4F	Farming and cattle rearing
Zargary Behak	3520	Subalpine forest	4	Lone	Islam	Shina	45	9M	Horticulture and cattle rearing
Gujjar Nar	3761	Alpine pasture	3	Bakarwal	Islam	Gojri & Hindko	74	9M/5F	Cattle rearing
Shounthar	3500	Subalpine forest	3.5	Kashmiri	Islam	Paharhe & Hindko	69	12M/6F	Farming and cattle rearing
Magri	3584	Alpine pasture	3.5	Kashmiri	Islam	Paharhe	82	13M/3F	Farming and cattle rearing
Ratti Galli	3814	Alpine pasture	3	Bakarwal	Islam	Gojri	77	8M/6F	Farming and cattle rearing
Dabran top	3352	Subalpine forest	3	Gujjar	Islam	Gojri	113	10M/3F	Farming and cattle rearing
Peera Hashimar	3079	Alpine pasture	4	Gujjar	Islam	Gojri & Hindko	106	8M/4F	Farming and cattle rearing
Sheesha Mali	3297	Alpine pasture	3	Gujjar	Islam	Gojri	72	6M/2F	Cattle rearing
Bara Hazari	3594	Subalpine forest	3.5	Kashmiri	Islam	Hindko	109	11M/4F	Cattle rearing
Machiyara	3423	Alpine pasture	4	Gujjar	Islam	Gojri & Hindko	98	8M/3F	Farming and cattle rearing
Kamiri top	3026	Alpine pasture	3	Maldiyal	Islam	Hindko	66	3M/5F	Cattle rearing
Sheero Dhara	2886	Alpine pasture	3	Gujjar	Islam	Gojri & Hindko	83	9M/2F	Farming and cattle rearing
Bedori	3082	Alpine pasture	3	Gujjar	Islam	Gojri & Hindko	62	6M/1F	Farming and cattle rearing
Poonch	2702	Alpine pasture	3.5	Bakarwal	Islam	Hindko & Gojri	70	4M/4F	Farming and cattle rearing
Ganga Choti	3024	Alpine pasture	3	Bakarwal	Islam	Hindko & Gojri	61	3M/4F	Farming and cattle rearing
Saral Behak	3621	Alpine pasture	3	Bakarwal	Islam	Gojri	74	7M/4F	Cattle rearing
Kutton	3423	Subalpine forest	3.5	Gujjar	Islam	Gojri	49	3M/3F	Farming and cattle rearing

*M* male, *F* female

### Floral diversity and anthropogenic pressure

**Fig. 3:**
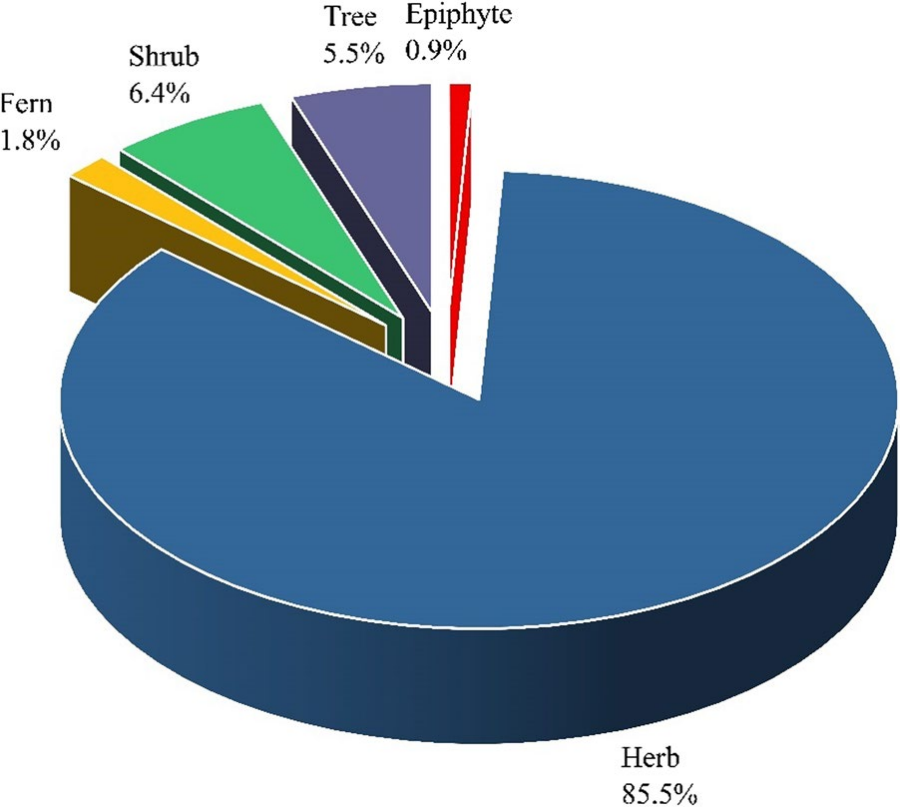
Classification of floral diversity on the basis of habit

**Fig. 4:**
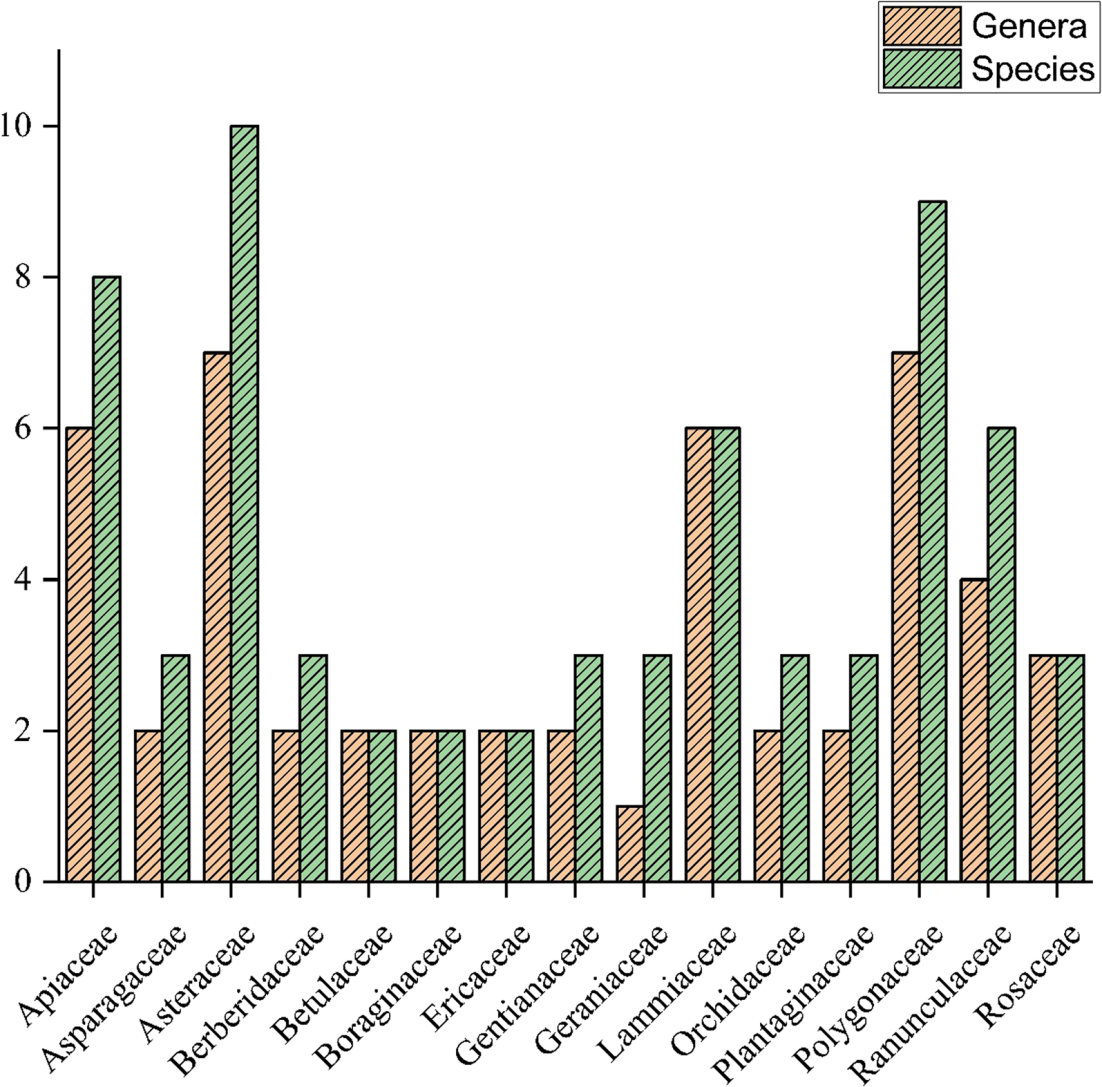
Proportion of dominated Plant Families and Genera recorded from the study area

The floral diversity of medicinal plants belonging to different families, local names, used parts, voucher numbers, ailments, remedies, and modes of administration are presented in Table 2. An ethnomedical plant survey documented 110 medicinal plants belonging to 90 genera and 49 families. Out of these medicinal taxa, herbs (85.45%) were predominant, followed by shrubs (6.36%), trees (5.45%), ferns (1.81%), and epiphytes (0.90%) (Fig. 3). Among the 49 families, Asteraceae was the dominant family and contributed the most (9.09%) to medicinal plants, followed by Polygonaceae (8.18%), Apiaceae (7.27%), Lamiaceae (5.45%), and Ranunculaceae (5.45%) (Fig. 4). The genera with the highest number of species represented are *Angelica* (3 species), *Geranium* (3 species), *Aconitum* (3 species), *Polygonatum* (2 species), *Taraxacum* (2 species), *Berberis* (2 species), and *Rheum* (2 species). The study shows that indigenous peoples of the Kashmir region heavily depend on medicinal plants for primary healthcare, especially during the winter when the area is inaccessible for longer due to heavy snowfall. These medicinal plant species were traditionally used to strengthen and energise, thereby improving health. In addition, the use of medicinal plants found at higher elevations is primarily driven by remoteness, poverty, and limited job opportunities. The study region's lack of infrastructure and socio-economic development exacerbates the problems. The survey revealed that high anthropogenic pressure on medicinal plants is likely caused by illegal extraction and environmental stress. Hence, local populations heavily depend on these higher elevation medicinal plants to sustain their primary healthcare and daily requirements. Asteraceae, Polygonaceae, Ranunculaceae, Apiaceae, Lamiaceae, and Ranunculaceae have been reported as dominant families from the Western Himalayas by several other researchers as well [38, 48–50]. The Asteraceae, Polygonaceae, and Ranunculaceae plant families are most prevalent in many open-habitat ecosystems [51, 52]. These aforementioned plant families are rich in medicinal chemical compounds such as sterols, alkaloids, glycosidase, and flavonoids, which are used for the treatment of numerous health problems [43]. These families were recorded as dominant families due to their broader ecological amplitudes and adaptations like small dwarf size, stunted growth, and semi-woody and spiny vegetation at higher altitudes with extreme environmental conditions [53]. Several other studies on ethnomedicinal applications in the Himalayan region support the present study results [54–57].

**Table 2:** Taxonomic diversity of plants used as ethnomedicines by the local communities of Kashmir

Sr. no	Plant name	Family	Voucher number	Local name	Parts used	UV	RFC	Ailments	Mode of preparation and administration
1	*Sambucus wightiana* Wall. ex Wight & Arn	Adoxaceae	EMP-10507	Ghinola	Ar, L	0.23	0.23	Wound healing	Paste made from the root is applied to the injured parts for wound healing
2	*Allium humile* Kunth	Amaryllidaceae	EMP-10443	Mali Da Pyaz	Wp	1.29	0.78	Stomach, gas trouble	30–40 gm of dried plant powder is used with 3–5 sips of warm water for 5 days and is also used as a flavouring agent in all dishes
3	*Angelica archangelica* L	Apiaceae	EMP-10446	Palhaar	R	0.50	0.50	Wound healing	Roots are crushed and then pasted on the wound site
4	*Angelica cyclocarpa* (C.Norman) M.Hiroe	Apiaceae	EMP-10447	Morchar	Rh	0.44	0.24	Influenza, cough, constipation, cold, asthma	20–40 gm of dried rhizome boiled in a cup of tea and taken before bed for 5–6 days
5	*Angelica glauca* Edgew	Apiaceae	EMP-10448	Chora	R, L	0.92	0.49	Stomach, acute abdominal pain, hepatitis rheumatism	Dried powder containing about 15–20 mg is used before breakfast, and a cup of tea made from 10–15 mg roots is used twice a week
6	*Bupleurum longicaule* Wall. & DC	Apiaceae	EMP-10456	kali jari	R	0.19	0.19	Headache	50 mg of crushed roots with a half glass of water are used to cure headaches twice a day
7	*Chaerophyllum villosum* Wall. & DC	Apiaceae	EMP-10458	Hasbay di Jar	Rh	0.30	0.23	Antibacterial, skin diseases, typhoid fever	Rhizomes have antifungal and antibacterial activity; a paste of the rhizome is given to cure typhoid fever and skin diseases
8	*Hymenidium brunonis* Lindl	Apiaceae	EMP-10479	Chorial	Rh	0.39	0.27	Influenza abdominal problems, cough, cold,	Root paste with sugar is used to cure the abdominal pain, cold, influenza, and cough of animals
9	*Hymenolaena candollei* DC	Apiaceae	EMP-10524	Palhaar	Wp	0.19	0.17	Fever	Fresh and dried plant powders are used for the treatment of fever
10	*Selinum vaginatum* C.B.Clarke	Apiaceae	EMP-10534	Mutakheshi	Wp	0.12	0.12	Cold	An extract of the whole plant is used directly on the chest and neck region for the treatment of colds
11	*Arisaema jacquemontii* Blume	Araceae	EMP-10449	Surm gaanda	R	0.45	0.40	Cough, General weakness intestinal	The middle part of the boiled rhizome is separated and cut into small pieces; 2–3 small pieces are taken daily
12	*Asparagus racemosus* Willd	Asparagaceae	EMP-10513	Sanspee	R	0.11	0.11	Fever	A cup of juice made from 15–20 mg of grinded root was used to cure fever twice a day for one week
13	*Polygonatum multiflorum* (L.) All	Asparagaceae	EMP-10525	Kawar Gandal	L, R	0.18	0.16	Cough, headache	5–10 mg of the dried rhizomes and leaves are mashed in a mortar, and then plant parts paste is used for the treatment of cough and headache
14	*Polygonatum verticillatum* (L.) All	Asparagaceae	EMP-10526	Kawar Gandal	L, R	0.14	0.14	Cough, fever	5–10 mg of the dried rhizomes and leaves are mashed in a mortar, and then plant parts paste is used for the treatment of cough and headache
15	*Achillea milliefolium* L	Asteraceae	EMP-10436	Dand juri	L, Rh	0.67	0.43	Stomach problems, toothache, urinary problems, antiseptic	Fresh leaves are chewed twice daily, mashed in mortar, and used directly on the aching or wound site
16	*Inula royleana* DC	Asteraceae	EMP-10468	Poshgar	R	0.28	0.25	abdominal problems, worm killing	Roots are soaked in water; an infusion is obtained and used early in the morning on an empty stomach for 6–8 days
17	*Dolomiaea macrocephala* DC. ex Royle	Asteraceae	EMP-10469	Gugal Dhoop	Rh	0.48	0.37	Joint pains, digestion, diarrhoea, backache,	Rhizome paste is used to cure digestion, backache, diarrhoea, and joint pains. Rhizome paste is eaten after being cooked in rice or flour
18	*Aucklandia costus* Falc	Asteraceae	EMP-10489	Kouth	Rh	0.52	0.43	Constipation, joint pain, antiseptic, toothache, body weakness, backache, worm killing	Dried rhizome (20–30 mg) is grinded into powder form. A half spoon of powder is taken mostly before breakfast on an empty stomach for 5–10 days. 3–4 pieces of rhizome are cooked with rice and used to cure backache and joint problems
19	*Ligularia jacquemontiana* (Decne.) M.A.Rau	Asteraceae	EMP-10490	Muta-khaesh	R	0.61	0.47	Gynae problems, gas trouble	Dried root powder, about 10–15 mg, is taken after meals to cure gas trouble in humans and is also used in the release of milk after birth in animals
20	*Taraxacum officinale* F.H.Wigg	Asteraceae	EMP-10492	Hand	L, R	0.27	0.22	Diabetes, cold, cough, kidney issues	0.5–1 kg of fresh leaves are cooked as vegetables to cure diabetes and kidney problems. 10–15 gm of dried root powder are used twice a day for one week to cure colds and coughs
21	*Taraxacum tibetanum* Hand. -Mazz	Asteraceae	EMP-10493	Bhoti Hand	Wp	0.26	0.18	Diabetes	0.5–1 kg of fresh leaves are cooked as a vegetable to cure diarrhoea twice a week
22	*Inula racemosa* Hook.f	Asteraceae	EMP-10505	Poshkara	R	0.21	0.17	Blood pressure, intestine problems	Roots oil, about 10–15 ml, is mixed with local dishes and used to lower blood pressure as well as stimulate peristalsis in the intestine
23	*Sonchus oleraceus* L	Asteraceae	EMP-10540	Hand	Wp	0.18	0.16	Wound healings, swellings	4–5 Drops of extract are used directly on the skin surface to cure wounds and swellings
24	*Taraxacum laevigatum* (Willd.) DC	Asteraceae	EMP-10544	Hand/Boti	R	0.39	0.31	Digestion, backache, joint pains, diarrhoea,	Root paste is used to cure digestion problems, backaches, diarrhoea, and joint pains. Root paste is eaten after being cooked in rice or flour
25	*Diplazium maximum* (D.Don) C.Chr	Athyriaceae	EMP-10535	Neeli Chaal	Wp	0.19	0.19	Body pain	20–30 mg of whole plant decoction is used to cure whole body pain; it is also used as a wild vegetable
26	*Berberis umbellata* Wall. ex G.Don	Berberidaceae	EMP-10452	Sumbal	R, B, F	1.04	0.56	Eye diseases, skin diseases, backache, piles, jaundice, joint pains, stomach problems, malaria, bone fractures	Root bark is soaked overnight in water; an infusion is obtained and used before breakfast for 20 days
27	*Berberis jaeschkeana* Schneider	Berberidaceae	EMP-10453	Kala Sumbal	B	0.44	0.21	Cancer, vertebral column pain, bleeding	100 gm of root bark is soaked overnight in water; an infusion is obtained and used for 10–15 days
28	*Podophyllum hexandrum* Royle	Berberidaceae	EMP-10508	Ban-khakri	L, R	0.23	0.18	Skin problems	Leaves and roots are crushed and applied for skin problems. Fruits are eaten raw
29	*Betula utilis* D.Don	Betulaceae	EMP-10533	Bhurji	Ra, L, B	0.40	0.22	Wounds, jaundice, haematuria, bone fracture	Extracted resins are used for the treatment of wounds; decoction of leaves is used for jaundice; and a wooden triangle is used for the treatment of haematuria (blood in the urine) in animals
30	*Corylus colurna* L	Betulaceae	EMP-10537	Jungli Doda	L	0.28	0.14	Fever, jaundice	A decoction of fresh leaves is used for the treatment of fever and jaundice
31	*Arnebia benthamii* (Wall. ex G.Don) I.M.Johnst	Boraginaceae	EMP-10450	Gow zaban	Rh	0.51	0.35	Fever, joint pain, fever, stomach-ache, ulcer	Crushed rhizomes are brewed in tea and used for 5–10 days
32	*Lindelofia longiflora* Baill	Boraginaceae	EMP-10471	Lhendi	Fl, R	0.24	0.24	Female diseases	Decoction of 10–15 gm of roots and flowers is used to cure female diseases for one month
33	*Capsella bursa-pastoris* Medik	Bracicaceae	EMP-10457	Pahn Pencha	Ar	0.27	0.17	Stomach	Aerial parts of the plant are cooked as a vegetable to cure stomach problems
34	*Amaranthus viridis* L	Chenopodiaceae	EMP-10445	Ganhiyar	Ar, Se	0.53	0.39	Backache, joint pain, stomach	Above ground, parts are used as vegetables, while seeds and other medicinal plants are used for 10 days
35	*Cuscuta reflexa* Roxb	Convolvulaceae	EMP-10510	Nela-thari	Wp	0.22	0.16	Jaundice, fever	40–50 gm of the whole plant is soaked in an open pot overnight, then water is used to cure jaundice and fever
36	*Rhodiola fastigiata* (Hook.f. & Thomson) Fu	Crrassulaceae	EMP-10486	Bagu Masti	Rh	0.46	0.38	Stomach, headache	15–30 mg of rhizome is soaked in the water overnight; an infusion is obtained and used on an empty stomach for 10 days
37	*Dioscorea deltoidea* Wall	Dioscoraceae	EMP-10459	Karens	Rh	0.17	0.11	Fever	5–10 gm of rhizome is soaked overnight in water; an infusion is obtained and used 2–3 times daily for 3–4 days
38	*Dryopteris filix-mas* (L.) Schott	Dryopteridaceae	EMP-10460	Langroo	Wp	0.58	0.45	Cholera, dysentery	Crushed whole plants are used for the treatment of cholera and dysentery
39	*Equisetum arvense* L	Equesetaceae	EMP-10461	Band kiya	Ar	0.25	0.18	Hepatitis, jaundice	Aerial parts are soaked in water; an infusion is obtained and used on an empty stomach early in the morning to cure hepatitis and jaundice
40	*Gaultheria trichophylla* Royle	Ericaceae	EMP-10517	Ferozi Boti	L, Fl	0.21	0.17	Forehead	The leaf and flower are mashed in a mortar, and the paste is applied to the forehead twice a week
41	*Rhododendron campanulatum* D.Don	Ericaceae	EMP-10529	Nychree	Fl, L	0.12	0.12	Muscular pain	Dried flowers and leaves Powder is mixed with oil (Desi ghee) and used for massage to cure muscular pain
42	*Euphorbia wallichii* Hook.f	Euphorbiaceae	EMP-10516	Jungli Dhodhal	R	0.12	0.12	Constipation	15–20 mg of dried root soaked in a cup of water is obtained and used for the treatment of severe constipation for 1–2 days
43	*Lotus corniculatus* L	Fabaceae	EMP-10522	Peela Phool	Wp	0.11	0.11	Skin surface	A 100 mg whole plant extract was applied directly to the skin's surface
44	*Quercus semecarpifolia* Sm	Fagaceae	EMP-10528	Mali da Choor	L	0.11	0.11	Gaseous bloat	1–1.5 kg of leaves is used for the treatment of gaseous bloat, especially in cattle
45	*Gentiana alii* (Omer & Qaiser) T.N.Ho	Gentianaceae	EMP-10465	Bhangrii	R	0.11	0.11	Leucorrhoea	Root extract is used to inhibit the pathogenic activity of yeast and is also used to cure lecoria
46	*Swertia petiolata* Royle	Gentianaceae	EMP-10499	Rech Endeh	Wp	0.38	0.35	Fever, stomach	4–5 young plants are grinded, and paste is used twice daily for 4–6 days
47	*Gentiana kurroo* Royle	Gentianaceae	EMP-10504	Bhangri	Wp	0.18	0.18	Blood purifier	Whole plant (40–70 gm) is dried, and the paste is used as a blood purifier
48	*Geranium nepalense* Sweet	Geraniaceae	EMP-10466	Ratan Jog	Wp	0.84	0.60	Gynae problems, toothache, throat, joint pain, constipation, digestion	Oil is astringent and applied as a massage around the throat. Decoction is used for the treatment of joint pain, gynaecological problems, constipation, and digestion
49	*Geranium wallichianum* D.Don	Geraniaceae	EMP-10538	Ratan Jog	Wp	0.57	0.49	Gynae problems, tonsillitis, toothache, joint pain, constipation, digestion, throat,	Oil is astringent and applied as a massage around the throat. Decoction is used for the treatment of joint pain, gynaecological problems, constipation, and digestion
50	*Geranium caespitosum* E.James	Geraniaceae	EMP-10539	Ratan Jog	Wp	0.58	0.45	Gynae problems, tonsillitis, toothache, throat, joint pain, constipation, digestion	Oil is astringent and applied as a massage around the throat. Decoction is used for the treatment of joint pain, gynaecological problems, constipation, and digestion
51	*Hypericum perfotatum* L	Hypericaceae	EMP-10511	Dudh-Jari	L	0.28	0.23	Anxiety, depression	A decoction of leaves is taken in cases of anxiety and depression as well as to boost the mood. Green tea is made by using 20–30 gm of dried leaves
52	*Iris hookeriana* Foster	Iridaceae	EMP-10519	Gahory Ghaa	R	0.32	0.28	Snake bites	5–10 mg of crushed root paste is used directly on body parts against snake bites 2–3 times
53	*Ajuga integrifolia* Buch. -Ham	Lammiaceae	EMP-10442	Jan-Adam	Wp	0.80	0.58	Skin diseases, diabetes, worm killing, blood purification, burning sensation of stomach	Decoction of 8–12 gm of crushed whole plant mixed with one glass of water and a small addition of sugar is given before breakfast (locally called Naroui Jarri)
54	*Mentha longifolia* (L.) L	Lammiaceae	EMP-10473	Podena	Wp	0.45	0.34	Digestion, diarrhoea, vomiting	Dried leaf powder 20–30 gm is taken with 3–4 sips of water to treat digestion and diarrhoea. Decoction of leaves is used to stop vomiting
55	*Origanum vulgare* L	Lammiaceae	EMP-10474	Ban Babri	Wp, Se	0.39	0.28	Skin diseases, fever, cough, intestinal, rheumatism	A decoction made by boiling 30–50 gm of fresh plant is taken 3–4 times daily to cure skin diseases, fever, cough, and rheumatism. Volatile oil is used to kill harmful intestinal worms
56	*Thymus linearis* Benth	Lammiaceae	EMP-10495	Ban jomair	L, Fl	0.73	0.55	Body shivering, urine, constipation	Decoction of 20–35 gm of leaves and flowers is used daily for 5–8 days
57	*Lamium flexuosum* Ten	Lammiaceae	EMP-10520	Sonhri Boti	Fl	0.17	0.17	Cough	A flower decoction is used for the treatment of cough, usually before going to bed in the evening
58	*Salvia hians* Royle	Lammiaceae	EMP-10530	Thandi juri	L	0.09	0.09	Eye disease	1–3 drops of fresh leaf extract are usually used against eye treatment, usually in the morning and evening until it cures
59	*Fritillaria cirrhosa* D.Don	Liliaceae	EMP-10464	Jungli thoom	Bu	1.34	0.70	Stomach, cancer	100 mg of dried roots are grinded and taken as a half spoon daily with 2–3 sips of water for one month
60	*Lilium polyphyllum* D.Don	Liliaceae	EMP-10521	Juri	Tu	0.13	0.13	Body pain	A decoction of 10–15 mg tubers is used for the treatment of body pain for 2–4 days in a week
61	*Alcea rosea* L	Malvaceae	EMP-10444	Gul khaar	R	0.29	0.22	Red urination, burning of feet, liver, stomach	Decoction of 50–70 gm crushed roots with half a glass of water is given before breakfast or after meals
62	*Malva parviflora* L	Malvaceae	EMP-10472	Dag Sonchal	Wp, Se	0.28	0.18	Cough, ulcers	1.5–2.5 kg of fresh, whole plant cooked as a vegetable to cure cough and ulcers in the bladder, for 2–3 days before bedtime, apply seed paste to the chest to relieve cold symptoms
63	*Trillium georgianum* S.B.Farmer	Melanthiaceae	EMP-10496	Tira-Patra	Rh, Ar	0.60	0.48	Heart diseases, rheumatism, joint pain, birth control, eye pain, blood cancer	Extracts of the rhizome and aerial parts have antifungal activity and are also used to cure rheumatism, practise birth control, and prepare sex-like hormones. They are also used to treat eye pain, joint pain, rheumatism, heart diseases, and blood cancer
64	*Habenaria pectinata* D.Don	Orchidaceae	EMP-10467	Nar-Mada	Rh	0.22	0.15	Fever	10–15 gm of crushed rhizome in a cup of tea are used for 2–3 days
65	*Herminium edgeworthii* (Hook.f. exCollett)	Orchidaceae	EMP-10518	Nar-Mada	Tu	0.11	0.11	Diabetes	Infusion of tubers used for the treatment of diabetes
66	*Dactylorhiza hatagirea* (D.Don) Soó	Orchidaceae	EMP-10542	Nar-Mada	Rh	0.07	0.07	Ringworm	10–15 gm of crushed rhizome in a cup of tea are used for 2–3 days
67	*Oxalis corniculata* L	Oxalidaceae	EMP-10506	Khatibooti	Ar, L	0.41	0.33	Hook worms, skin problems	An infusion of aerial parts (5–10 mg) is given to children for the removal of hook worms. Fresh leaves are eaten raw for their flavour and to stimulate the salivary glands. Leaves are crushed with onions to obtain a half cup of juice, which is used to treat skin warts
68	*Meconopsis aculeata* Royle	Papaveraceae	EMP-10523	Kant	Fl	0.10	0.10	Fits	5–8 gm of flowers are soaked in water overnight and then used orally twice a week for the treatment of fits in children
69	*Corydalis govaniana* Wall	Papaveraceae	EMP-10543	Peela Phool	L	0.18	0.13	Joint pian, joint swelling	An extract of 10–15 gm of fresh leaves is used around the joints before going to bed in the evening, twice a week
70	*Phytolacca acinosa* Roxb	Phytolaccaceae	EMP-10476	Lubbar	Ar, R	0.21	0.14	Infections, swellings, joint pains, wounds, weight loss, asthma, dysentery	1–1.5 kg of fresh or dried aerial parts are cooked as vegetables; 20–25 mg of crushed root paste is taken with warm water for 1 day a week
71	*Abies pindrow* Royle	Pinaceae	EMP-10435	Rever/Tung	B, Ra	1.05	0.38	Fever, cough, stomach pain	Bark is boiled in tea (Qehwa) and used for one week, 2–3 times daily
72	*Plantago lanceolata* L	Plantaginaceae	EMP-10478	Chamchi Patr	Fl, L	0.30	0.22	Dysentery	Fresh leaves cooked as vegetables; flowers as an alternate to ispaghol in dysentery
73	*Plantago major* L	Plantaginaceae	EMP-10512	Chamchi Patar	L, R	0.30	0.22	Snake bite, feet healing	Paste made from the root is applied to the skin as an antidote for snakebite. 15–25 mg of fresh leaves are crushed and applied on feet for healing the heel. Leaves are cooked as vegetables
74	*Wulfeniopsis amherstiana* (Benth.) D.Y.Hong	Plantaginaceae	EMP-10532	Booti	L	0.15	0.13	Fever, headache	Decoction of fresh leaves is used for the treatment of headache and fever
75	*Aconogonon alpinum* (All.) Schur	Polygonaceae	EMP-10439	Pan cholla	Ar, R	0.35	0.28	Leucorrhoea	Extract of 5–8 mg of crushed roots is used with a half glass of water
76	*Persicaria amplexicaulis* (D.Don) Ronse Decr	Polygonaceae	EMP-10455	Mali di Masloonr	Rh, F	0.63	0.48	Weakness, diarrhoea, stomach problem, dysentery, haemoptysis,	Roots are used for general weakness and for the treatment of diarrhoea, dysentery, and haemoptysis. Dried flower, 10–15 gm in tea, is used to treat stomach problems
77	*Fagopyrum esculentum* Moench	Polygonaceae	EMP-10462	Tarumba	L	0.31	0.22	Constipation, weakness	Fresh leaves are collected and cooked as vegetables for the treatment of diarrhoea and constipation
78	*Oxyria digyna* Hill	Polygonaceae	EMP-10475	Khat-kurla	Ar	0.31	0.22	Stomach, constipation	1–2 kg of fresh aerial parts are cooked as vegetables for stomach problems and constipation
79	*Rumex alpinus* L	Polygonaceae	EMP-10480	Chikron	Ar, R, Se, S	0.51	0.43	Pain relief, constipation, ulcers	1 kg of aerial parts are cooked as vegetables. Seeds are used for relieving the gripping pain of colic. Root paste is used as an astringent. Stalk is soaked in water, and an infusion is obtained and used 3–4 times daily for 2–3 days to cure ulcers and constipation
80	*Polygonum aviculare* L	Polygonaceae	EMP-10481	Masloon	Rh	0.28	0.23	Antibacterial activity, stomach	70–80 gm Dried rhizome tea is used twice a day for several weeks
81	*Rheum australe* D.Don	Polygonaceae	EMP-10484	Goal Chotial	L. Rh, S	0.85	0.55	Dysentery, muscular injury, wounds, headache, mumps, stomach, constipation, swelling of throats, blood purification	1–2 kg of fresh aerial parts are cooked as vegetables; 50–60 mg of rhizome are ground into a paste and taken early in the morning before breakfast or twice a day for one week
82	*Rheum webbianum* Royle	Polygonaceae	EMP-10485	Chapti Chotial	L, Rh, S	0.53	0.44	Dysentery, muscular injury, wounds, headache, mumps, stomach, earache, constipation, swelling of throats, blood purification	1–2 kg of young stem of plant are cooked as a vegetable, and 20–40 mg of root paste is applied externally to muscular injuries, cuts, and wounds for 5–10 days
83	*Rumex nepalensis* Spreng	Polygonaceae	EMP-10488	Hola	L, Rh	0.21	0.14	Constipation, anti-lice	2–3 kg of young leaves are collected and then air dried for 10–15 days. In the winter, they are cooked as vegetables to cure constipation in humans and animals. Root paste is used as an anti-lice agent
84	*Primula denticulata* Sm	Primulaceae	EMP-10483	Mamera	L, Wp	0.27	0.17	Eye diseases, urination	Extract of 25–40 40 gm of fresh leaves mixed with water is used to cure eye diseases. A whole plant decoction of 70–100 mg is used for the treatment of urination problems in animals
85	*Primula macrophylla* D.Don	Primulaceae	EMP-10527	Tareri Paatar	Fl	0.18	0.18	Cough, fever	Powder made from dried flowers is used for the treatment of fever and cough for one week
86	*Caltha palustris* L	Ranuanculaceae	EMP-10514	Pani Patrr	L	0.17	0.17	Menstrual disorder	Dried leaf powder is fried in oil (ghee) and then used for the treatment of female menstrual disorders for 5–8 days
87	*Clematis napaulensis* DC	Ranuanculaceae	EMP-10515	Boty-bail	Fl	0.16	0.16	fever	A cup of juice extracted from 50–70 mg of fresh leaves is used for the treatment of fever for 2–3 days
88	*Aconitum hetrophyllum* Wall	Ranunaculaceae	EMP-10437	Patrees	Rh	0.88	0.68	Pneumonia, cold, fever, headache, diarrhoea, dyspepsia, diabetes	Rhizome paste is applied to the chest, and 5–8 gm of rhizome boiled in a cup of tea is used twice a day for five days
89	*Aconitum violaceum* Jacquem. ex Stapf	Ranunaculaceae	EMP-10541	Patrees	Rh	0.43	0.39	Pneumonia, fever, headache and diabetes	A cup of tea made with 10–15 gm of rhizome mixed with other medicinal plants is taken for one week
90	*Aconitum chasmanthum* Stapf	Ranunculaceae	EMP-10438	Mohree	Fl, Rh	0.80	0.60	Asthma, mukhar in animals	1–2 mg of dried flowers mixed with honey, then a spoonful of honey used twice a day for 15 days
91	*Actaea spicata* L	Ranunculaceae	EMP-10440	Muniree	R, F,	0.28	0.18	Anti-lice	Decoction of roots and fruit was used for one week
92	*Fragaria nubicola* Lindl	Rosaceae	EMP-10463	Khan merchant	F, R	0.64	0.44	Jaundice, typhoid	Fruit eaten as a strawberry, a decoction of roots weighing about 30 gm is used to cure jaundice and typhoid
93	*Potentilla atrosanguinea* Lodd., G.Lodd. & W.Lodd	Rosaceae	EMP-10482	Mali di cha	R	0.41	0.34	Stomach	To treat stomach problems, a cup of tea made from 10 to 15 gm of root mixed with other medicinal plants is consumed
94	*Rosa webbiana* Wall. ex Royle	Rosaceae	EMP-10487	Gulab	Fl	0.23	0.17	Massage in pain	Decoction of flowers (50–70 mg) is used for massage to cure body pain
95	*Skimmia laureola* (DC.) Decne	Rutaceae	EMP-10491	Neera	L	0.68	0.55	Obesity, insect repellent, dysentery, cough, body strengthening	Decoction of leaves was used to reduce obesity. A 5–10 mg paste is mixed with honey to cure coughing and taken before going to bed in the evening. Tea is also used for strengthening the body
96	*Aesculus indica* (Wall. ex Cambess.) Hook	Sapindaceae	EMP-10441	Bun Khor	F	0.30	0.20	Body weakness	Fruit is used twice a week
97	*Acer caesium* Wall. ex Brandis	Sapindaceae	EMP-10503	Tra-kana	Ar	0.29	0.15	Stomach	5–10 mg of leaf paste is used to cure stomach problems; wood is used for making furniture and is also used as fuel, to make utensils, and as agricultural tools
98	*Bergenia stracheyi* (Hook.f. & Thomson) Engl	Saxifragaceae	EMP-10454	Bat Phewa	Rh	1.20	0.54	Burns, ulcer dysentery, kidney stone, diabetes, piles, heart diseases, backache, obesities	Root paste is used for the cure of burns, kidney stones, diabetes, ulcers, and heart disease. 50 gm of crushed roots are mixed with milk for the cure of backache. Dried root powder mixed with water or milk is used to reduce obesity
99	*Bergenia ciliata* (Haw.) Sternb	Saxifragaceae	EMP-10536	Bat Phaewa	Rh	0.44	0.18	Burns, ulcer dysentery, kidney stone, diabetes, piles, heart diseases, backache, obesities	Root paste is used on burns, in kidney stones, piles, diabetes, ulcer dysentery, and heart diseases. Crushed roots are mixed with milk and given as a backache. A half spoon of root powder with water or milk is taken on an empty stomach to cure stomach problems
100	*Lagotis cashmeriana* Rupr	Scrophularaceae	EMP-10470	Kali hand	Ar	0.60	0.49	Diabetes	Tea made from aerial parts 15–20 gm of plant is taken twice a day for 3–4 days
101	*Verbascum thapsus* L	Scrophularaceae	EMP-10501	Gady-Kan	L, Fl	0.31	0.23	Relaxation, wound healing, cold, dysentery, dye	250–400 mg of dried leaves is smoked to induce relaxation (locally known as Hukka or Chilam). A paste of 10–20 gm of leaves is applied externally for wound healing. Tea made from dried leaves is given to cure the common cold and dysentery. Flowers are used as dye by crushing them in water
102	*Picrorhiza kurrooa* Royle	Scrophulariaceae	EMP-10477	Koohr	Rh	0.33	0.29	Fever, jaundice, urinary, asthma, diabetes, cough, leukoderma, burning sensation	200 mg of dried roots are grinded into powder; a half spoon of powder is taken twice a day with milk, lassi, or water for 20–30 days
103	*Atropa acuminata* Royle ex Lindl	Solanaceae	EMP-10451	Lubar	L, R	0.28	0.18	Cough, fever, pyrexia	Decoction of leaf and root is used for the treatment of cough and fever
104	*Taxus wallichiana* Zucc	Taxaceae	EMP-10494	Thoonri/Burmi	L, B	0.85	0.29	Antiseptic, asthma, epilepsy, bronchitis, cough	Leaf extracts are sedative and antiseptic. 40–50 mg of bark tea is used to cure asthma, cough, epilepsy, and bronchitis
105	*Urtica dioica* L	Urticaceae	EMP-10502	Kayari	L, R	0.28	0.17	Diuretic, anti-allergic, wounds healing	20–50 gm Roots are crushed, and paste is applied to cuts and wounds for 4–5 days. Leaves are used as diuretics and anti-allergens. Paralysed parts of the body are treated by applying paste to the affected parts
106	*Valeriana jatamansi* Jones	Valirianaceae	EMP-10497	Musk-e-Bala	Rh	0.23	0.17	Mental disorders, fever, insecticide, joints pain, eye, ear, pneumonia	20–30 gm of rhizome paste is used daily for 5–6 days; an extract of rhizome is used externally as a massage around joints for 5 days, mostly before going to bed in the evening
107	*Valeriana himalayana* Grubov	Valirianaceae	EMP-10531	Mushka Baala	R	0.13	0.13	Fever, headache	Crushed roots are used for the treatment of fever and headache
108	*Viburnum grandiflorum* Wall. ex DC	Viburnaceae	EMP-10500	Ikloo	Wp, Fl	0.57	0.28	Blood purifier	A glass of juice extracted from 200–400 gm of fruit is taken as a blood purifier for 3–4 days; fruits are eaten raw; the whole plant is used as fuelwood; shoot branches are used as ropes
109	*Viola canescens* Wall	Violaceae	EMP-10498	Banafsha	Fl	0.25	0.20	Earache, jaundice, hepatitis	For one week, a 10–15 gm decoction of fresh flowers is mixed with sugar 1–2 times
110	*Viola biflora* L	Violaceae	EMP-10509	Banafsha	L, Fl	0.25	0.22	Malaria	A decoction of the entire plant mixed with sugar is given to Malaria patients or anyone suffering from a fever for any reason. Crushed leaves and flowers are given as vegetables to cure liver problems

*UV* use value, *RFC* relative frequency citation, *Wp* whole plant, *Ra* resin, *B* bark, *L* leaf, *R* root, *Fl* flower, *Ar* aerial part, *Rh* rhizome, *Se* seed, *F* fruit, *T* tuber, *Bu* bulb, *S* stem

### Endemic species

Endemic species refers to species found exclusively in a specific geographical area or specific habitat [58]. There were 31 endemic species in the study area, 15 of which were endemic to the Kashmir region and 16 to the Western Himalaya. *Arnebia benthamii*, *Bergenia ciliate, Bergenia stracheyi, Bupleurum longicaule, Caltha palustris, Corydalis govaniana, Dolomiaea macrocephala, Euphorbia wallichii, Gentiana alii, Lagotis cashmeriana, Ligularia jacquemontiana, Meconopsis aculeata, Podophyllum hexandrum, Rheum webbianum,* and *Taraxacum laevigatum* were found to be endemic to the Kashmir region*. Aconitum chasmanthum, Aconitum hetrophyllum, Aconitum violaceum, Aucklandia costus, Dactylorhiza hatagirea, Gentiana kurroo, Hymenidium brunonis, Hypericum perfotatum, Inula royleana, Iris hookeriana, Persicaria amplexicaulis, Polygonatum verticillatum, Rosa webbiana, Thymus linearis, Verbascum Thapsus,* and *Viola biflora* were found to be endemic to the Western Himalayan region*.*

### Observations

**Fig. 5:**
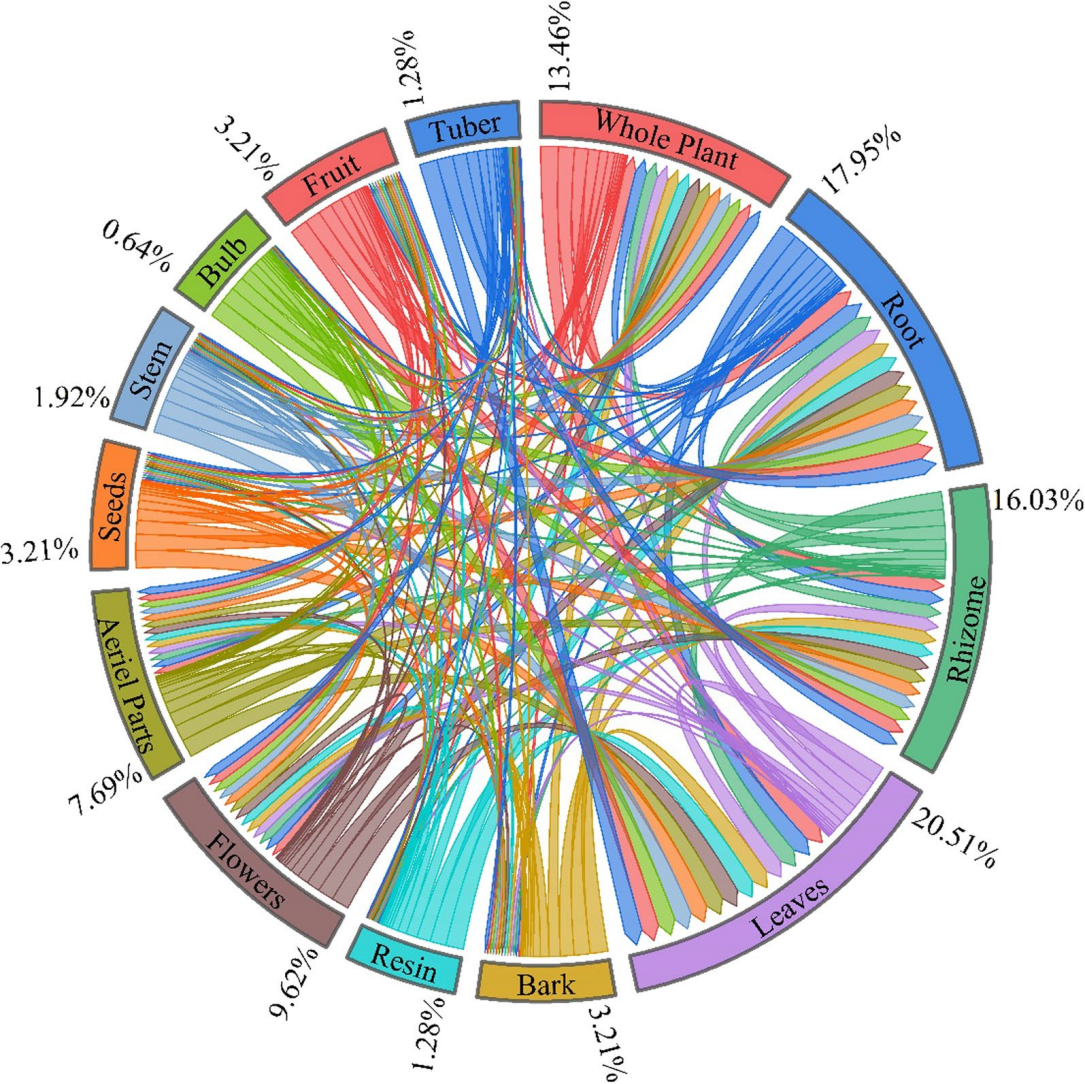
Proportion of plant parts used by medicinal plants for remedies

**Fig. 6:**
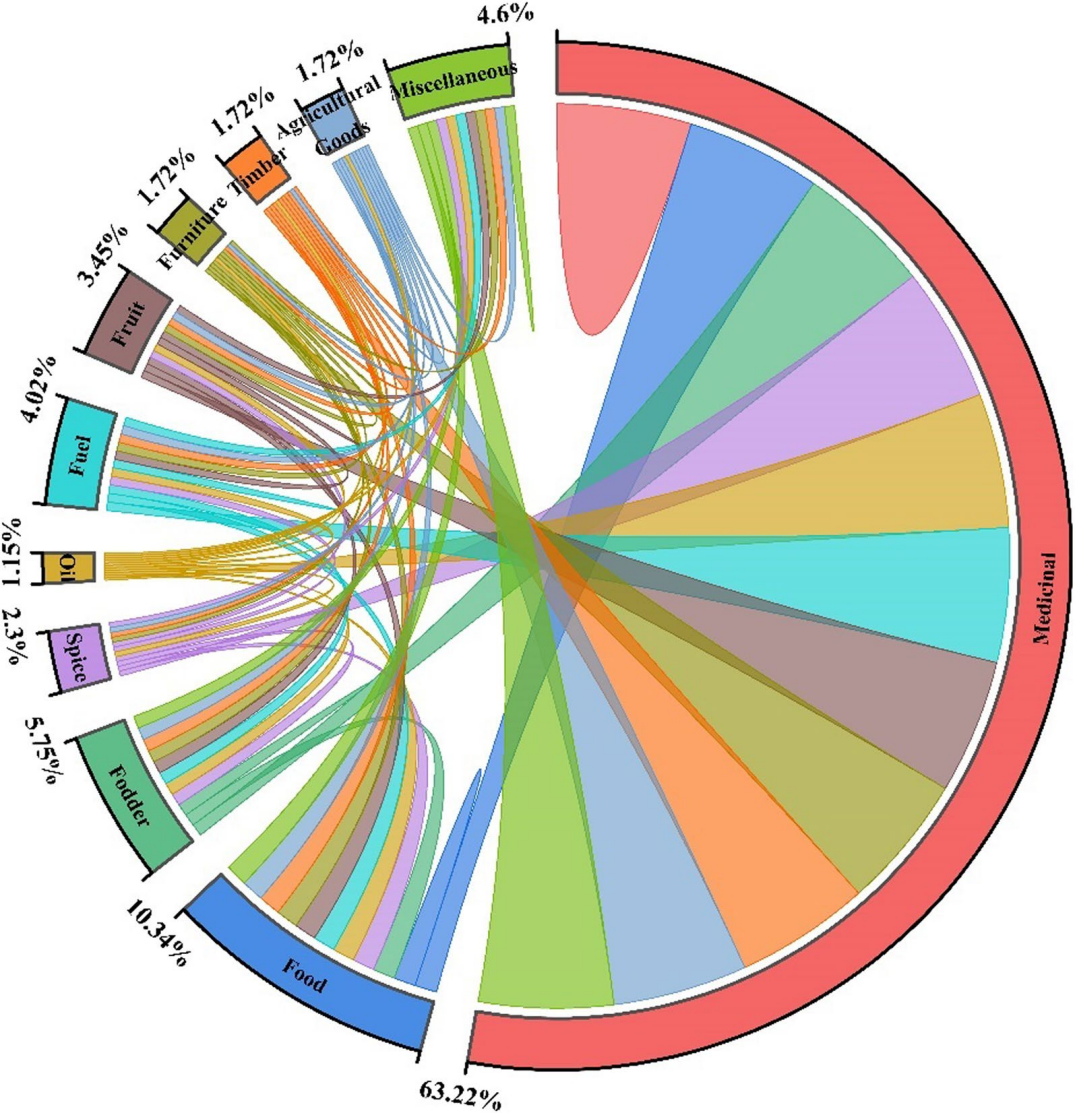
Percentage distribution of medicinal flora on the basis of use category

A total of 166 remedies were used for the treatment of various diseases in humans, and 9 remedies were used for animals*. Quercus semecarpifolia* was a medicinal plant used only in veterinary medicine. Twelve medicinal plants (i.e. *Phytolacca acinose*, *Hymenidium stellatum*, Primula *denticulate*, *Rheum webbianum*, *Rumex nepalensis*, *Ligularia jacquemontiana*, *Verbascum Thapsus*, *Sambucus wightiana*, *Iris hookeriana*, and *Betula utilis*) were used for the treatment of both animals and humans. Thirteen medicinal plants (i.e. *Geranium wallichianum*, *Geranium caespitosum*, *Bergenia ciliate*, *Aucklandia costus*, *Rheum webbianum*, *Rheum austral*, *Picrorhiza kurrooa*, *Phytolacca acinose*, *Geranium nepalense*, *Bergenia stracheyi*, *Berberis umbellate*, *Ajuga integrifolia*, and *Aconitum hetrophyllum*) were observed to have more diversified uses for the treatment of more than 5 ailments. All recorded species are wild and mostly collected from nearby subalpine forests and alpine pastures. Among the medicinal plants, herbs (85.5%) were most frequently used by the local populations of Kashmir (Fig. 3), whereas leaves (20.51%) were used for the treatment of various ailments (Fig. 5). Use category results for documented plants showed that the majority of plants were used for medicinal purposes (63.62%), followed by food (10.34%) and fodder (5.75%) (Fig. 6). Furthermore, females had better knowledge about medicinal plants and remedies for the treatment of different ailments because the majority of male members are out of their homes for jobs due to poverty and limited job opportunities. Similar findings were also reported from the Western Himalaya and Brazil [52, 59].

### Preparation of medicinal plants remedies and usage

**Fig. 7:**
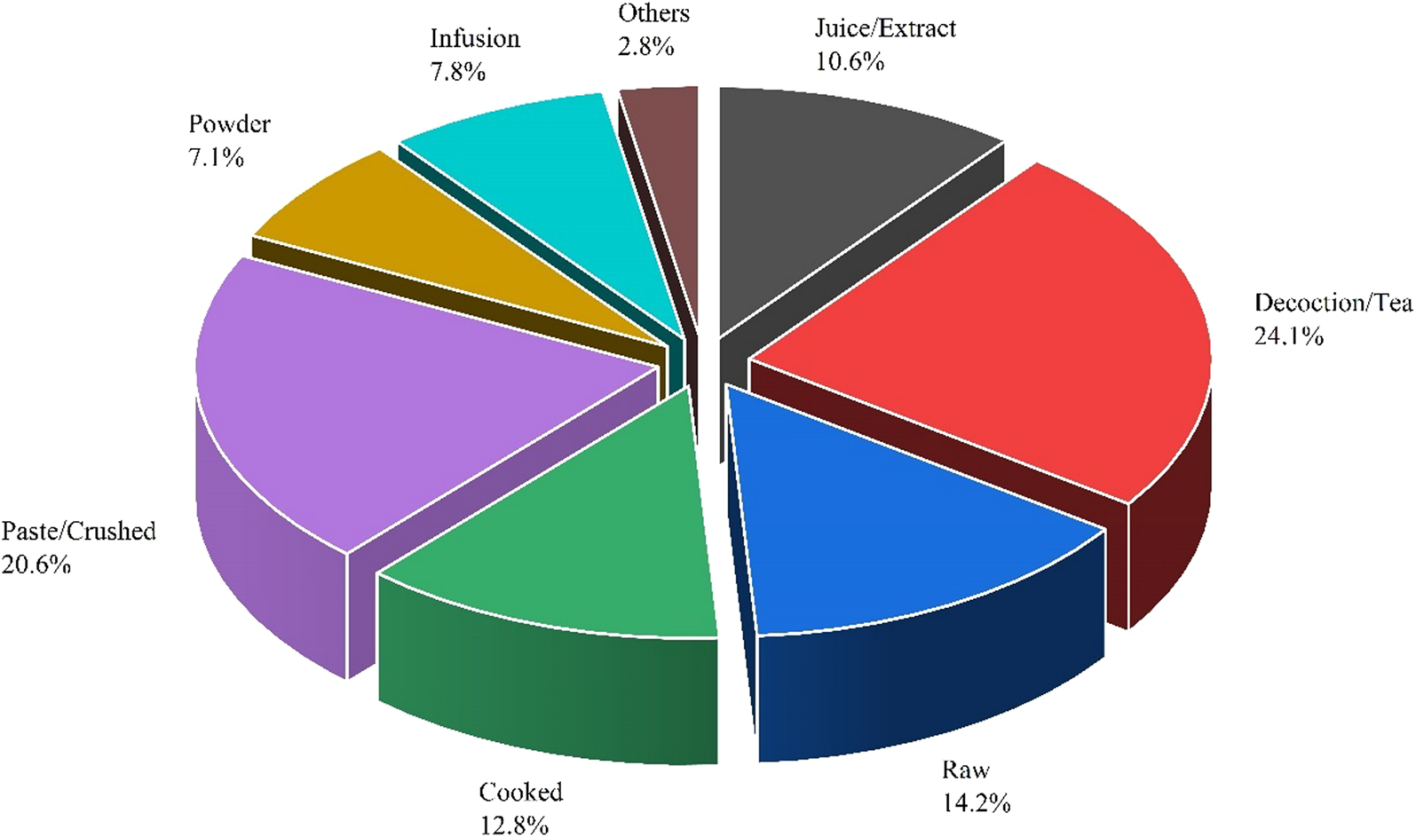
Percentage distribution of methods of remedies preparation

All parts of medicinal plants were used to prepare various remedies, such as leaves, rhizomes, roots, tubers, and stems. Herbs were recorded as the dominant life form (94 species) used as a major remedy source in the Kashmir region's local populations. The most frequently used medicinal remedies were tea or decoction (24.1%), paste or crushing (20.6%), raw (14.2%), cooked (12.8%), juice or extract (10.6%), infusion (7.8%), powder (7.1%), and others (2.8%) (Fig. 7). The present study results revealed that leaves (20.51%) were used for the preparation of various remedies, followed by roots (17.95%), whole plant (13.46%), rhizomes (16.03%), aerial parts (7.69%), the flowers (9.62%), bark (3.21%), seeds (3.21%), fruits (3.21%), stem (1.92%), resin (1.28%), tubers (1.28%), and bulbs (0.64%) (Fig. 5). Local communities residing in the mountainous region of Kashmir did not frequently use medicinal plants' bark, seed, stem, and bulbs, rendering these plant parts unsustainable for medicinal use. The aforementioned plant parts are difficult to crush for decoction and hence cannot sustainably be used as remedies in the local communities. The plant's parts, such as the root, entire plant, and rhizome, are extensively employed in native healthcare systems for basic healthcare purposes in Kashmir. So, these plant parts have been considered sustainable for medicinal use in the prevailing area as they contain major constituents of medications. Excessive use of leaves and roots, especially for vulnerable species, could adversely affect their growth, leading to population decreases and even species extinction [56]. Due to the presence of alkaloids, leaves can be used as food as well as a potential source of valuable pharmaceuticals [3]. As a result, they are frequently used as a remedy in the traditional healthcare system because they are so effective. A total of 166 remedies were made, of which 142 employed fresh parts of plants and the rest were made from 24 dried plant parts. Approximately 31.81% of the 110 species enlisted were used for the treatment of a specific type of disease. The most common methods of administration are oral (72.81%), external (24.56%), and eye drops (0.7%). The dosage and duration of medications are based on the severity of the disease, the age of the patient, and the information provided by different informants. Similar findings were also reported, which supports our results [52, 60–64]. The differences in the duration and dosage of medicinal plants are given in Table 2.

### Ailments treated

**Fig. 8:**
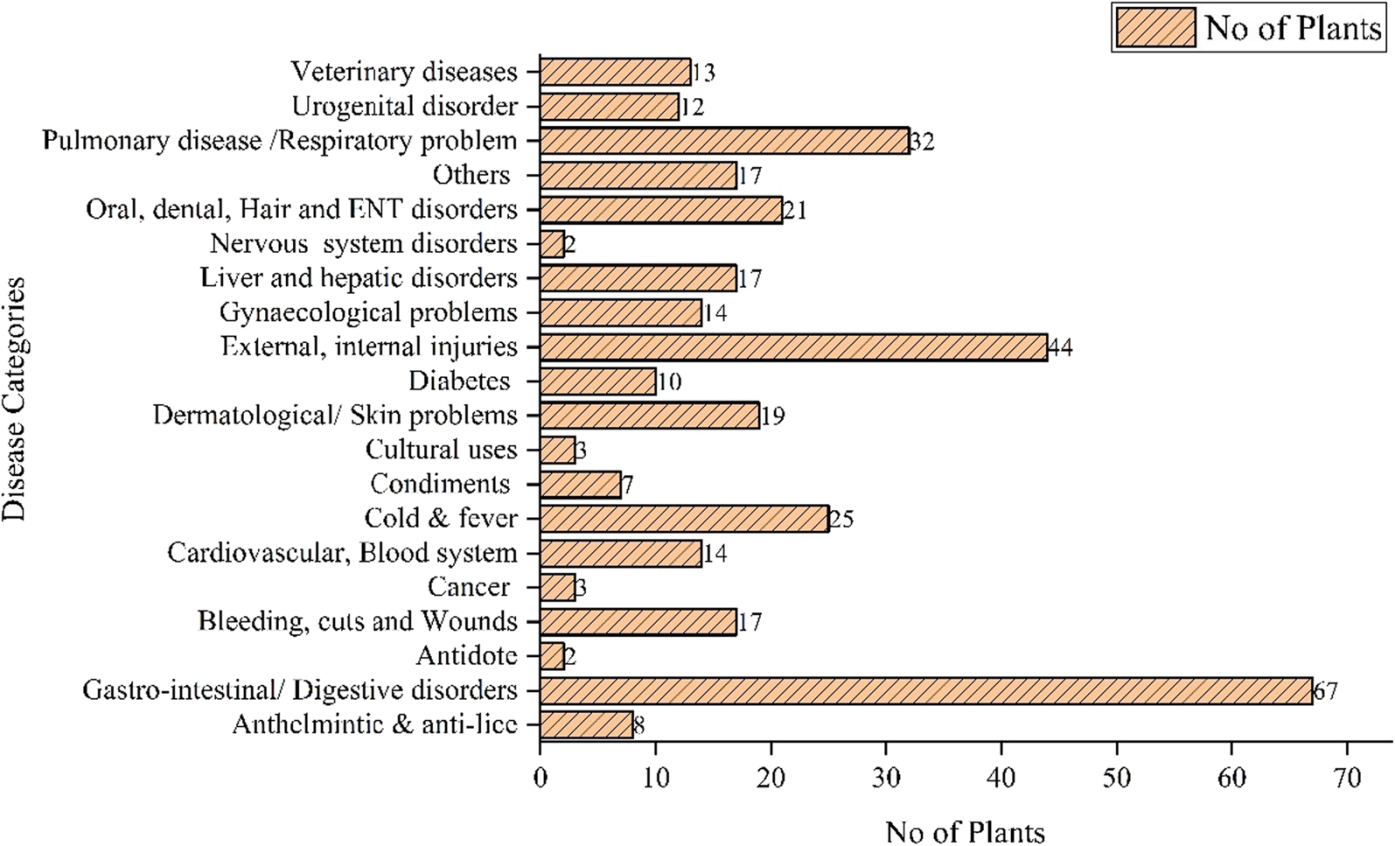
Major disease treated through medicinal plants

The most frequently recorded diseases from the Kashmir region are fever, stomach, body pain, coughs, colds, joint problems, constipation, wounds or cuts, and skin problems. Due to heavy snowfall in certain regions, local people have moved from high-altitude areas (i.e. alpine pastures) to lower elevations. In some areas, the availability of modern medications for quick pain relief has caused a decline in the use of medicinal plants. In contrast, the majority of people in remote areas still rely on medicinal plants for their primary healthcare. The documented ailments were classified into 20 general categories: gastro-intestinal/digestive disorders (67 remedies), external and internal injuries (44 remedies), pulmonary disease/respiratory problems (32 remedies), cold and fever (25 remedies), oral, dental, hair, and ENT disorders (21 remedies), dermatological/skin problems (17 remedies), bleeding, cuts, and wounds (17 remedies), liver and hepatic disorders (17 remedies), others (17 remedies), cardiovascular blood system (14 remedies), gynaecological problems (14 remedies), urogenital disorder (12 remedies), diabetes (10 remedies), anthelmintic and anti-lice (8 remedies), condiments (7 remedies), veterinary diseases (4 remedies), cancer (3 remedies), cultural uses (2 remedies), nervous system disorders (2 remedies), and antidote (2 remedies) (Fig. 8). Most studies reported ethnomedicinal applications for the treatment of gastroenterological, respiratory, and wound healing, which were recorded as the most prevalent diseases in the Himalayan region [4, 22, 62, 65, 66].

### Informants consensus factor

Different ailments reported from the study area were classified into 20 general categories to develop a consensus among informants (Fig. 8). The informant’s consensus factor was calculated based on disease categories, as single medicinal taxa are used for the treatment of 2–3 or more disease categories. The ICF value ranged from 0.64 to 0.85. The highest value of ICF was recorded for gastro-intestinal/digestive disorders (0.85), whereas the lowest value of ICF was recorded for liver and hepatic disorders (0.64). Among the categories, gastro-intestinal/digestive disorders were the leading disease categories based on ICF values, as strongly supported by various studies [67–73]. The prevalence of gastro-intestinal/digestive disorders is mainly due to fuel wood smoke inhalation unhygienic conditions, contaminated food, and poor water quality mostly arise in the study area due to animals and humans frequently sharing water sources, such as little springs and wells [67, 74, 75]. *Fritillaria cirrhosa*, *Allium humile*, *Bergenia stracheyi*, *Berberis umbellate*, *Angelica glauca*, *Ajuga integrifolia*, and *Achillea milliefolium* were the most commonly used medicinal plants for the cure of gastro-intestinal and digestive disorders. These medicinal plants were preferred to cure digestive disorders because they are a rich source of vitamins, essential oils, flavonoids, and other bioactive compounds [76].

The second highest categories of ICF were estimated for dermatological/skin problems (0.84). Skin problems might arise due to UV exposure at higher elevations, unhygienic living conditions, sharing a room with family members or even cattle, and contaminated food [77, 78]. The third highest IFC value (0.83) was recorded for the cold and fever categories. Cold and fever problems arise due to different factors, including harsh winter conditions such as frosting and snowfall, increased moisture at higher elevations, unhygienic conditions, and abrupt changes in weather [79]. The high ICF value (0.82) for gynaecological disorders in the study area may be due to limited access to hospitals and medicines, remoteness, and heavy snowfall causing road blockages. However, over 95% of females give birth naturally, without regular check-ups or operations. This is attributed to their healthy lifestyle, daily walking for daily needs, and reliance on medicinal herbs with no side effects.

### Use value

Use value is an essential tool for identifying highly valuable medicinal plants in a region for detailed pharmacological research. The use value ranged from 0.07 to 1.34 (Table 2). The highest use value was reported for *Fritillaria cirrhosa* (1.34) followed by *Allium humile* (1.29), *Bergenia stracheyi* (1.20), *Abies pindrow* (1.05), *Berberis umbellata* (1.04), *Angelica glauca* (0.92), *Aconitum hetrophyllum* (0.88), *Rheum australe* (0.85), *Taxus wallichiana* (0.85), and *Geranium nepalense* (0.84). The high usage of reported medicinal plants implies a strong association and reliance of local populations on the surrounding flora, particularly for the treatment of various diseases [43]. There is a strong correlation between use value and use reports for a plant. Medicinal plants with a higher number of records have a higher use value [67, 80].

### Relative frequency citation

RFC is a tool used for assessing the significance of different species in curing different ailments based on how often they are mentioned by local communities [36]. The RFC value ranged from 0.07 to 0.78 (Table 2). The highest RFC value was reported for *Allium humile* (0.78), followed by *Fritillaria cirrhosa* (0.70), *Aconitum hetrophyllum* (0.68), *Geranium nepalense* (0.60), *Aconitum chasmanthum* (0.60), *Ajuga integrifolia* (0.58), *Berberis umbellata* (0.56), *Rheum australe*, *Thymus linearis*, and *Skimmia laureola* (0.55), respectively. Locals are familiar with numerous plant species with high RFC values that are prevalent throughout the area due to their traditional use as medicine. These plant species could prove valuable for drug discovery and commercial authentication [70, 81]. High RFC values indicate that a species is commonly used for the treatment of various ailments, leading to overexploitation and a serious threat to conservation. Some plants with high RFC are critically endangered in the study area.

### Novelty of study

The findings of this study were compared with the 20 published papers from the Himalayan region represented in Table 3. In the comparison, the JI value ranged from 18.23 to 3.05 (Table 3). The maximum value of the Jaccard index was reported from District Poonch, Jammu & Kashmir (18.23), followed by the northern region of Kashmir, Himalaya (16.45), and District Chamba, Western Himalaya (16.27). High JI values indicate that the vegetation in these areas has similar characteristics due to common traditions, cultures, geography, and environmental conditions. The lowest value of JI was calculated in the Ladakh region of the Himalayas (3.05). The low JI value represents that the geographical features are different. The lower value of JI indicates that the high-elevation areas where the study was conducted do not possess similar topography to the cold desert of Ladakh, which accounts for the lowest value observed. It demonstrates that there are significant variations in the site features between the two regions, which is the reason the current study's JI value is the lowest.

**Table 3:** Jaccard Index data for medicinal plants

Investigated area	Region	SY	NOP	NORP	NOPSU	NOPDU	TSCBA	SEAA	SEOSA	POPSU	POPDU	JI	C
Rajouri and Poonch	J&K, India	2015	91	104	11	22	33	71	77	12.09	21.15	18.23	[82]
Kashmir	J&K, India	2022	300	67	7	18	25	42	85	2.33	26.87	16.45	[83]
Chamba	Western Himalaya	2019	135	83	10	17	27	56	83	7.41	20.48	16.27	[84]
Kashmir	Western Himalaya, India	2022	111	82	8	15	23	59	87	7.21	18.29	13.61	[85]
Jhelum valley	AJK, Pakistan	2021	152	113	10	16	26	87	84	6.58	14.16	13.20	[52]
Kashmir	Western Himalaya	2021	109	32	6	10	16	16	94	5.50	31.25	12.70	[56]
Gulmarg	J&K, India	2021	54	60	6	11	17	43	93	11.11	18.33	11.11	[86]
Bheri, Muzaffarabad	AJK, Pakistan	2016	300	80	8	9	17	63	93	2.67	11.25	9.83	[87]
AJK	AJK, Pakistan	2018	255	73	5	11	16	57	94	1.96	15.07	9.58	[63]
Kathua	J&K, India	2015	112	197	11	13	24	173	86	9.82	6.60	8.48	[88]
Poonch	Western Himalaya	2021	58	31	3	8	11	20	99	5.17	25.81	8.46	[89]
Poonch	J&K, India	2021	58	31	1	9	10	21	100	1.72	29.03	7.63	[90]
Kupwara	J&K, India	2018	60	23	4	5	9	14	101	6.67	21.74	7.26	[91]
Diamir	Western Himalaya, Pakistan	2022	166	61	5	5	10	51	100	3.01	8.20	6.21	[92]
Buner	Pakistan	2017	90	80	4	7	11	69	99	4.44	8.75	6.15	[93]
Budgam	J&K, India	2021	63	82	5	4	9	73	101	7.94	4.88	4.92	[94]
Dhirkot	AJK, Pakistan	2019	74	140	4	7	11	129	99	5.41	5.00	4.60	[79]
Pir Nasoora	AJK, Pakistan	2015	155	104	3	6	9	95	101	1.94	5.77	4.39	[95]
Deosai Plateau	Western Himalaya, Pakistan	2015	71	50	2	3	5	45	105	2.82	6.00	3.23	[22]
Ladakh	Trans-Himalaya, India	2022	105	25	1	3	4	21	106	0.95	12.00	3.05	[96]

*SY* study year, *NOP* number of plants, *NORP* number of reported plants, *NOPSU* number of plants with similar uses, *NOPDU* number of plants with different uses, *TSCBA* total species common in both areas, *SEAA* species enlisted in aligned areas, *SEOSA* species only enlisted in the study area, *POPSU* percentage of plants with same uses, *POPDU* percentage of plants with different uses, *JI* Jaccard index, *C* citation

This study reports 22 medicinal plants that are new or rarely documented in ethnomedicinal literature: *Aconogonon alpinum*, *Actaea spicata*, *Angelica archangelica*, *Asparagus racemosus*, *Berberis jaeschkeana*, *Bupleurum longicaule*, *Chaerophyllum villosum*, *Clematis napaulensis*, *Corylus colurna*, *Diplazium maximum*, *Gentiana alii*, *Herminium edgeworthii, Lindelofia longiflora*, *Polygonatum multiflorum*, *Potentilla atrosanguinea*, *Rhodiola fastigiata*, *Salvia hians*, *Selinum vaginatum*, *Taraxacum tibetanum*, *Taraxacum laevigatum*, *Viola biflora*, and *Valeriana himalayana*. This research discovered new ethnomedicinal uses of plants, i.e. the flower and rhizome of *Aconitum chasmanthum* for treating asthma in humans and mukhar in animals, the aerial part of *Aconogonon alpinum* for leucorrhoea, *Actaea spicata* as an anti-lice, roots of *Alcea rosea* for urination problems (red urine), roots of *Angelica archangelica* for wound healing, *Asparagus racemosus* for fever, *Berberis jaeschkeana* for cancer, bleeding, and vertebral column pain, *Bupleurum longicaule* for headache, *Chaerophyllum villosum* for typhoid fever and skin problems, *Clematis napaulensis* for fever, *Corylus colurna* for jaundice, *Diplazium maximum* for body pain, *Gentiana alii* for Leucorrhoea, *Herminium edgeworthii* for diabetes, *Geranium caespitosum* for gynae problems, *Gentiana kurroo* for blood purification, *Lindelofia longiflora* for female disorders, *Polygonatum multiflorum* for cough and headache, *Potentilla atrosanguinea* for stomach, *Rhodiola fastigiate* for stomach, *Salvia hians* for eye diseases, *Selinum vaginatum* for cold, *Taraxacum tibetanum* for diabetes, *Taraxacum laevigatum* for joint pain, *Valeriana himalayana* for fever and headache, and *Viola biflora* for malaria.

### Threats to medicinal plants

The indigenous communities in the mountainous areas of Kashmir rely on domesticated animals, agriculture, and natural resources for their livelihood. Due to remoteness and limited job opportunities, local communities rely on natural resources to sustain their lives at higher elevations. The majority of the aforementioned communities are involved in numerous activities, including deforestation, illegal wood smuggling, and the extraction of medicinal herbs and fungi (*Morchella esculenta*) as their main source of income. Unfortunately, over 50% of populations collect medicinal plants to sell to local herb sellers, resulting in the overexploitation of these medicinal plants and subsequently leading to the extinction of endemic medicinal plants. Numerous factors, such as natural disasters like avalanches and soil erosion, as well as anthropogenic activities like deforestation, urbanisation, forest fires, and overgrazing, are significant contributors to the decline of medicinal plants. To prevent these alarming trends, relevant authorities must implement effective conservation measures to ensure sustainable utilisation and protection of these important medicinal plants. Furthermore, the government, academic institutions, and the Forest Department's work to raise awareness about the importance, conservation, and cultivation of medicinal plants among the local community might serve as an important factor in restoring the declining numbers of these invaluable plants. These initiatives are anticipated to enhance the region's sustainability while simultaneously providing the foundation for its socio-economic development.

### Ecological transition and sustainability of medicinal plants

Biodiversity in the mountains is ecologically valuable because it regulates the stability of soil and is essential for the functioning of ecosystems, as well as having substantial social, ethical, and aesthetic values. Mountains comprise approximately 25% of the world's terrestrial biodiversity and have a presence in 50% of the world's biodiversity hotspots [97]. However, anthropogenic activities have caused remarkable changes in ecosystems over the last few decades, resulting in an alarming decline in biodiversity. The maintenance of a healthy and sustainable ecosystem is significantly challenged by the alarming rate of change driven by the human race, which is exceptional in human history. The impacts of the global climate change scenario have exerted a profound impact on different countries all over the world, contributing to a variety of ecological transformations such as glacial melting, altered precipitation patterns, droughts, floods, and a significantly increased worldwide temperature. Due to the instability of ecological processes, biodiversity is lost and ecosystems are degraded.

The significant contribution of wild medicinal plants for the treatment of different ailments in indigenous communities is currently under threat. Mountainous ecosystems, where a majority of medicinal plants grow, are particularly vulnerable to the adverse impacts of climate change due to their confined geographical boundaries. However, urbanisation, immense population pressure, expansion of agricultural fields, overgrazing, deforestation, forest fires, and overexploitation pose an additional threat to wild medicinal plants. The aforementioned factors have substantially decreased the diversity of medicinal plants that are readily available in mountainous areas. The extraction of wild medicinal plant rhizomes, roots, and flowers by untrained local collectors has imposed enormous stress on their populations, leading to the extinction of many endangered and endemic taxa. This represents a significant threat to the availability of basic healthcare in remote regions that depend significantly on the healing properties of wild medicinal plants due to a lack of modern healthcare facilities.

In the context of climate change, ecological transition is a serious and debatable issue around the world, and policy makers are tasked with implementing effective strategies to protect against the increasing risk of extinction and ensure the uninterrupted availability of wild medicinal plants for local communities. As a consequence, immediate action is required to ensure the sustainable long-term utilisation and preservation of these valuable natural resources. There are 400 endemic species in Pakistan, and the northern and western higher elevations of Pakistan and Kashmir are home to 80 per cent of the country's endemic flowering plants [98]. Mountainous ecosystems are characterised by their rich biodiversity because they are home to a variety of protected areas and an extensive diversity of medicinal plants as well as animals. Due to the existence of endemic medicinal plants, the conservation of mountainous ecosystems is of utmost significance [99]. Hence, it is critically important that decision-makers give thoughtful consideration and then implement measures that prioritise the conservation and sustainable uses of these valuable resources, ensuring their availability for future generations.

The active participation of doctors in indigenous communities is one way to encourage the use of wild medicinal plants for basic healthcare. This comprises educating and promoting awareness among local communities and patients about the potential healing properties and health advantages of wild medicinal plants. Doctors who are familiar with the traditional usage of wild medicinal plants can be highly valuable in promoting their use in basic healthcare. They may guide their patients on how to properly and successfully include natural remedies in their treatment plans while urging them to consider these practices. However, doctors’ involvement in encouraging the therapeutic potential of medicinal plants incorporates the opportunity to have a significant impact on local populations by improving healthcare access and promoting sustainable utilisation of natural resources.

### Importance of revitalising sustainable ethnomedicinal practices

In isolated mountainous areas, indigenous knowledge is disappearing or being replaced by non-native knowledge, which is a serious problem. Youth education is critical for the sustainable utilisation of local natural resources and the preservation of cultural heritage. Traditional knowledge is dwindling due to social changes and reliance on standardised plant elements, threatening the viability of local practises. Due to the availability of modern medication and modern agricultural methods, traditional ecological knowledge in mountainous areas is being lost. Due to the aforementioned problems, some of the medicinal plants are rare, and a few are overexploited. In the future, this could have a severe negative impact on basic healthcare practices. Numerous indigenous communities are losing traditional foraging practices as a result of socio-environmental changes. The majority of wild medicinal plants are commonly available, and LEK needs to be revitalised to foster the ecological transition. Ethnobotanical research documenting the disappearance of wild plant knowledge can be an invaluable tool to prepare for future challenges.

Ethnobiologists have historically emphasised the crucial need to revitalise the valuable traditional ethnomedicinal knowledge. Ethnobiologists demand the development of dynamic frameworks that not only revive these old traditional healthcare practises but also encourage their implementation through holistic educational approaches [100, 101]. The revitalisation of traditional nature, ethnomedicinal knowledge, and healthcare practises are essential for achieving socio-ecological sustainability. Rural communities are facing inevitable social change; modern medication availability, driven by advancement in the pharmaceutical industry, is erasing traditional healthcare practices. Documentation of traditional knowledge from field investigations may provide concrete tools for better understanding and future planning in numerous fields. The participation of ethnobiologists in policy platforms and community engagement should be strongly encouraged since they play a critical role in understanding and communicating how humans significantly interact with nature. Incorporating regionally oriented biological learning into educational curricula that emphasise local healthcare management along with modern medications in pharmaceutical fields by involving students could be crucial for fostering sustainability. This strategy, proposed by Pontius [102], encourages students to investigate and compare traditional practises with scientific findings and to actively participate in the co-creation of a sustainable future. Integrating traditional healthcare management knowledge into modern education systems can be a strong tool for strengthening students understanding of culture, traditional healthcare practises, nature, and their environment, as well as promoting creative connections with their socio-ecological environments. Field expeditions that encourage connection with nature through interaction, compassion, emotion, meaning, and beauty might be beneficial in improving our relationship with the natural world [103]. To learn about healthcare management through medicinal plants effectively, it is essential to implement reflective and multilateral methodologies that include both belief based and practical aspects of traditional knowledge.

Ethnoecological knowledge about medicinal plants can be valorised by numerous approaches, such as the preservation of traditional knowledge: It is critical to preserve traditional collection and preparation methods to safeguard indigenous herbal history. This can be accomplished through the documentation of indigenous knowledge and urging the usage of local medicinal plants for basic healthcare to sustain their continued existence. Education: the appreciation of traditional herbs can be fostered by imparting knowledge of their cultural significance through seminars and other cultural events. Promoting local healthcare practises: promoting indigenous healthcare practises through newspapers, social media, and herbal festivals can raise awareness regarding their cultural significance and encourage residents to try them out. Promoting Mountainous Tourism: Indigenous medicinal practices can help the tourism industry by attracting tourists who are interested in traditional cuisine and herbal remedies. Increasing awareness of herbal remedies can benefit the local economy. Innovation: By investigating novel uses of traditional herbal remedies, innovation can help preserve folk plant medicine. This includes experimenting with traditional foods or incorporating traditional herbal teas into modern cuisine. Furthermore, valuing indigenous healthcare practises entails understanding the cultural significance of the local medicinal value of plants and taking steps to safeguard and foster them for future use.

## Conclusion

The study presents the first detailed exploration of indigenous ethnomedicinal knowledge from remote mountainous areas at higher elevations in Kashmir, Western Himalayas. The mountainous region of Kashmir is home to diverse wild medicinal plants and traditional knowledge, both of which play a significant role in treating various ailments through primary healthcare. The study emphasises the importance of indigenous ethnomedicinal knowledge as well as the declining interest in gaining traditional knowledge among the younger generation, possibly due to an increase in allelopathic medicinal practices. Kashmir's mountainous floral diversity is threatened by overexploitation, illegal smuggling, overgrazing, soil erosion, and deforestation. These factors led to the extinction of important wild, endemic medicinal plants. Local communities are mostly unaware about the importance and conservation of medicinal plants. The documentation of indigenous knowledge is essential for its preservation, sharing of information in the public domain, the invention of novel medicines, and future management for the conservation of threatened flora.

## Data Availability

All data are available in this paper.

## Abbreviations

LEK: Local ecological knowledge
AJK: Azad Jammu and Kashmir
RFC: Relative frequency of citation
UV: Use value
ICF: Informant consensus factor
JI: Jaccard index
