# Correction: The local medicinal plant knowledge in Kashmir Western Himalaya: a way to foster ecological transition via community-centred health seeking strategies

**DOI:** 10.1186/s13002-024-00654-3

**Published:** 2024-02-19

**Authors:** Muhammad Manzoor, Mushtaq Ahmad, Muhammad Zafar, Syed Waseem Gillani, Hamayun Shaheen, Andrea Pieroni, Abdullah Ahmed Al-Ghamdi, Mohamed Soliman Elshikh, Saddam Saqib, Trobjon Makhkamov, Khislat Khaydarov

**Affiliations:** 1 Department of Plant Sciences, Quaid-i-Azam University, Islamabad, Pakistan; 2 State Key Laboratory of Plant Systematics and Evolutionary Botany, Institute of Botany Chinese Academy of Sciences, Beijing, China; 3 Department of Botany, University of Azad Jammu & Kashmir, Muzaffarabad, 13100 Pakistan; 4 University of Gastronomic Sciences Pizza V. Emanuele II, 12042 Pollenzo, Bra, Italy; 5 Department of Botany and Microbiology, College of Sciences, King Saud University, Riyadh, Saudi Arabia; 6 University of Chinese Academy of Sciences, Beijing, China; 7 Department of Forestry and Landscape Design, Tashkent State Agrarian University, 2 A., Universitet Str., Kibray District, 100700 Tashkent Region, Uzbekistan; 8 Institute of Biochemistry, Samarkand State University, Samarkand, Uzbekistan; 9 Department of Medical Analysis, Tishk International University, Erbil, 44001 Kurdistan Iraq

**Correction: Journal of Ethnobiology and Ethnomedicine (2023) 19:56** 10.1186/s13002-023-00631-2

Following publication of the original article, it was brought to the journal's attention that affiliation 5 was incorrectly detailed. Affiliation 5 has since been corrected in the article to the following: Department of Botany and Microbiology, College of Sciences, King Saud University, Riyadh, Saudi Arabia.

The authors thank you for reading this erratum and apologize for any inconvenience caused.

